# Optimization of triazole-based small molecule disruptors of the Aha1/Hsp90 complex that manifest potent activities

**DOI:** 10.1016/j.ejmech.2026.118612

**Published:** 2026-02-02

**Authors:** Kevin C. Catalfano, Margaret H. Rakonick, Brian S.J. Blagg

**Affiliations:** Department of Chemistry and Biochemistry, Warren Center for Drug Discovery, The University of Notre Dame, 305 McCourtney Hall, Notre Dame, IN, 46556, USA

**Keywords:** Hsp90, Aha1, Small molecule disruptors, Triazole

## Abstract

Recent structure-activity relationship (SAR) studies identified multiple Hsp90/Aha1 small molecule disruptors that manifest moderately improved potencies and physiochemical properties. Among these molecules, a 1,5-bisubstituted-1,2,3-triazole containing molecule (Triazole A), demonstrated the importance of a *cis*-amide bond. Consequently, this work aimed to optimize Triazole A via a nitrogen scan and Topliss tree approach. The potency was determined against cell lysates and co-Immunoprecipitation (co-IP) experiments. The most efficacious molecules were evaluated for physiochemical properties that include aqueous solubility, human liver microsome half-life, and MDCK-MDR1 permeability. New molecules emerged from this study that manifest nanomolar potency and improved activity in cell-based co-IP experiments. These new molecules represent a significant improvement over prior Hsp90/Aha1 disruptors and provide chemical tools to investigate the significance of Hsp90/Aha1 complexes.

## 1. Introduction

The 90 kDa heat shock proteins (Hsp90) are ubiquitously expressed ATP-dependent molecular chaperones that are responsible for the three-dimensional maturation, stabilization and degradation of >400 client proteins [1,2]. Among them are receptors, kinases and intrinsically disordered proteins including tau, which is known to aggregate and drive disease progression in Alzheimer's Disease and other neurodegenerative diseases called tauopathies [3]. Many of these disease states lack effective therapeutic interventions. To address this unmet need, researchers developed Hsp90 N-terminal ATP-competitive inhibitors. Unfortunately, nearly all of the inhibitors failed clinically due to on-target and dose-escalating toxicities related to pan-inhibition [4,5]. To overcome these detriments, researchers shifted their focus to disrupting the interactions between Hsp90 and co-chaperones, which are proteins that bind Hsp90 to regulate the protein folding cycle. Activator of Hsp90 ATPase activity homolog 1 (Aha1), is the most potent stimulator of Hsp90 ATPase activity. Imbalances in the protein folding machinery lead to aberrant Hsp90 activity that drives disease states including cancer and neurodegeneration, which can be facilitated by ATP-stimulation [6,7].

Recent studies demonstrated that Aha1 overexpression in the hippocampus of rTg4510 tau transgenic mice led to increased tau aggregation and neuronal death. Our group further demonstrated that KU-177, a coumarin containing molecule based on the Hsp90 C-terminal inhibitor novobiocin, can disrupt Hsp90/Aha1 interactions without inhibition of Hsp90 client maturation [7]. Retaining Hsp90 protein folding function is important to treat tauopathies because free Hsp90 can fold misfolded tau. Unfortunately, KU-177 exhibits poor drug-like properties. Therefore, SAR studies were performed that replaced the coumarin core with a substituted phenyl ring and demonstrated the key pharmacophore is the central amide moiety [8]. To investigate this further, a series of amide bioisosteres were prepared and found to exhibit improved activities and drug-like properties (Fig. 1). In this manuscript, SAR analyses were performed on the triazole isostere, which led to small molecules that manifest nanomolar activities and improves physiochemical properties.

## 2. Results and discussion

### 2.1. Synthesis and in vitro evaluation of Hsp90/Aha1 disruptors

Past work focused on developing bioisosteric replacements for the central amide moiety of KU-177, which demonstrated that *cis*-amide bioisosteres were preferred. Among the more efficacious molecules, the 1,5-bisubstituted triazole, triazole A, was chosen as a lead for this study. Nitrogen scans and chlorinated derivatives were prepared to improve activity as shown in Schemes 1-4. Initial derivatives of ring A were synthesized by a Suzuki coupling reaction between iodide **1** and boronic acid **2** to yield aniline **3**. The aniline underwent diazotization to form azide **4**, which was used in Ru-catalyzed alkyne azide cycloaddition (RuAAC) reactions with various ethynylbenzenes (**5a-5f**, Scheme 1). Ring B was derivatized according to Schemes 2-4. Pyridinyl methyl ester **7** (see Scheme 2)was used in a Suzuki-coupling reaction with boronic acid **2**. The resulting biaryl methyl ester **8** was hydrolyzed to afford carboxylic acid **9**, which was converted to acid chloride **10**. The acid chloride was reacted with 4-trifluoromethyl aniline (**11**) to afford amide **12**, which was converted to tetrazole **13**. The tetrazole was chosen as a surrogate for the triazole due to synthetic challenges associated with 2-pyridinyl azides in RuAAC [12].

3-Pyridinyl and chlorinated derivatives were accessed via Scheme 3. Anilines **14a-d** were subject to Suzuki-coupling reactions with boronic acid **2**, then biaryl anilines **15a-d** were converted to the corresponding azides **16a-d**. Each azide was subject to RuAAC with 4-trifluoromethyl ethynylbenzene (**17**) to give triazoles **18a-d**. 4-Pyridinyl triazole **22** was accessed via Scheme 4, wherein 4-amino-2-bromo pyridine (**19**) was converted to the corresponding azide **20**. The azide was then reacted with **17** in RuAAC to afford triazole **21**. Then, **21** was used in a Suzuki-coupling reaction with boronic acid **2** to give triazole **22**.

Ring C was derivatized according to Scheme 5. Aniline **1** was converted to azide **23**, followed by a RuAAC reaction with ethynylbenzene **17** to yield triazole **24**. Triazole.

**24** was reacted with various boronic acids (**25a-f**) in Suzuki-coupling reactions to afford pyridinyl and chlorinated triazoles **26a-f**. Pyridinyl and chlorinated derivatives of each phenyl ring were evaluated for their potency to disrupt Hsp90/Aha1 interactions via a split *renilla* luciferase assay (Tables 1-3). Derivatives of ring A exhibited significant improvement in potency, especially compound **6e**, wherein the *m*-pyridine exhibited mid nanomolar potency (Table 1). The lead triazole (Triazole A) manifests an IC_50_ of 4.60 ± 0.4 μM.

B ring derivatives were evaluated and shown to exhibit low activity or no activity at 5 μM, which suggests that alterations to this ring do not improve potency (Table 2). This may present an opportunity to attach solubilizing groups or modifications useful as chemical tools (e.g. biotin). The final derivatives from the nitrogen scan and chlorinated derivatives as well as their potencies are shown in Table 3. Chlorinated derivatives exhibited improvements in activity, and **26b** was shown to manifest sub-micromolar.

potency. However, the greatest improvement in potency was observed via the nitrogen scan. It is notable that the potency improved and was dependent upon the substitution pattern of the pyridine where *p > m > o*.

Initial biological evaluation revealed additional SAR studies were necessary. The nitrogen scan results in Table 3 demonstrated significantly improved potency via the *para* and *meta* pyridine (**26e-f**). Therefore, new derivatives were synthesized according to.

Scheme 5 with the boronic esters below (Fig. 2). These derivatives integrate unsaturated oxygen-containing heterocycles that replace in lieu of the *p*-pyridine to demonstrate if the nitrogen atom is necessary for improved potencies. These derivatives are shown in Table 4 (**26g-26n**). The 3,4-pyridazine **26g** was prepared to hybridize the activity from **26e** and **26f**, however it was inactive at 5 μM. Among the oxygen containing derivatives, dihydropyran **26h** exhibited improved activity, while **26i** resulted in a loss of potency, which suggested that the nitrogen atom was not necessary, and that a heteroatom at the 4-position was preferred. Therefore, the 4-methoxy derivative **26l** was evaluated and exhibited improved potency. To better understand the importance of ring size, **26m** and **26n** were prepared. Unfortunately, they manifested weaker activity, which indicated that the 6-membered ring is preferred. However, compound **26k** lost potency 10-fold, which suggests that increased steric bulk is also disfavored.

In the interest of investigating fully saturated heterocycles, products **26h** and **26n** underwent Pd/C-catalyzed hydrogenation to afford products **27** and **28** (Scheme 6). However, evaluation of **27** and **28** demonstrated that the double bond present in **26h** and **26n** is necessary for activity. Derivatization of product **26j** upon treatment with HCl yielded tetrahydropyridine **29**, which was formylated and acetylated to give **30** and **31** respectively (Scheme 7). Surprisingly, **29** was inactive, which indicated that the oxygen or a hydrogen bond acceptor is preferred. In fact, **30** and **31** were inactive despite the presence of the carbonyl oxygen, which can participate in hydrogen bonding. It is possible that small changes led to conformational adjustments that are disfavored within the binding site. An additional oxygen-containing saturated heterocycle, morpholine **32** was accessed via Cu-catalyzed N-arylation of triazole **24** as shown in Scheme 8, but was also found to be inactive at 5 μM. Together, these results demonstrate that the double bond in **26h** is necessary for activity and that oxygen is preferred over nitrogen at this position. Given the high potency of **26l** and the pyridine derivatives, **37a** and **37b** were synthesized according to Scheme 9 by triflation of **33** to give **34**. Subsequent Miyaura borylation of **24** afforded phenylboronic ester **35**, which was reacted with triflate **34** and 4-bromopyridine **36** to give **37a** and **37b** respectively. Surprisingly, spirocycle **37a** produced decreased potency, but **37b** exhibited near equipotency to compound **26.**

Further examination of the methoxy substituent on **26l** led to the synthesis of alcohol **41**, which can serve as both a hydrogen bond donor and acceptor. Alcohol **41** was prepared according to Scheme 10. Hydroxyl boronic ester **38** was subject to TIPS protection, then intermediate **39** was reacted with **24** in a Suzuki-coupling reaction to give **40**, which was deprotected to afford **41**. Surprisingly, **41** was inactive, which suggests the methoxy is necessary as a hydrogen bond acceptor. Compound **26l** exhibits good potency, but the methoxy will be readily metabolized. Therefore, alcohol **41** was used in an electrophilic trifluoromethoxylation reaction with the Togni reagent II to yield compound **42** [14]. Unfortunately, **42** was inactive, which may be attributed to a less electron dense oxygen due to the electron withdrawing CF_3_ group.

Since pyridine **5e** and dihydropyran **26h** exhibit good activity individually, hybridized molecule **44** was prepared to synergize their activity according to Scheme 11. Synthesis of molecule **44** began with RuAAC of ethynylpyridine **5e** and azide **23** to give triazole **43**. Intermediate **43** was subject to a Suzuki-coupling reaction with **25h** to furnish triazole **44**. Unfortunately, **44** did not manifest enhanced potency, but instead maintained activity equipotent to **5e** with an IC_50_ of 0.15 ± 0.01 μM suggesting that potency for this molecule is driven by the A ring (Scheme 11).

### 2.2. In cellulo and physiochemical evaluation of Hsp90/Aha1 small molecule disruptors

Following *in vitro* evaluation, we aimed to demonstrate whether these derivatives could disrupt Hsp90/Aha1 *in cellulo* at lower concentrations without inducing the heat shock response or client degradation associated with Hsp90 inhibition (Fig. 3). Co-Immunoprecipitation (co-IP) experiments with SH-SY5Y cells demonstrated effective disruption of Hsp90/Aha1 following 24-h treatments with doses of 0.3 and 0.1 μM for **26f** and **26h**, respectively (Fig. 3A). Notably, 24-h treatments with doses of 0.3 and 0.1 μM for **26f** and **26h**, respectively (Fig. 3A) did not result in Cdk6 degradation nor induction of Hsp70 expression in SH-SY5Y cells, but the Hsp90 pan-inhibitor 17-AAG did induce these effects (Fig. 3B), which supports our hypothesis that these disruptors do not directly inhibit the Hsp90 protein folding process at these doses. Additional co-IP experiments were performed in SK-Br-3 cells, where **26f** and **26h** successfully disrupted Hsp90/Aha1 at lower doses than the lead triazole (Fig. 3C, Supporting Fig. 1). Triazoles **26l** and **37b**, among the most potent compounds that emerged in this study, were evaluated for physiochemical properties. Compound **26l** exhibited improved solubility, permeability and ER as compared to triazole A, but had a short half-life as expected (Table 5). Whereas compound **37b** exhibited improved solubility, half-life, and a low ER. In addition, **37b** exhibits a reduced molecular weight, a good tPSA and low cLogP. Collectively, **37b** exhibits the greatest potency and most favorable physiochemical profile compared to other potent compounds that emerged in this study and represents a promising pre-clinical candidate.

## 3. Conclusion

This work aimed to develop a new scaffold for Hsp90/Aha1 disruption via amide bioisosteric replacement. We successfully improved the potency of the lead 1,5-bisubstituted-1,2,3-triazole (triazole A) greater than 100-fold, which represents the most potent Hsp90/Aha1 disruptors disclosed to date. Compounds **26f** and **26h** manifest improved activity *in vitro* and successfully disrupted Hsp90/Aha1 complexes *in cellulo* in both in SH-SY5Y neuroblastoma and SK-Br-3 breast cancer cell lines without induction of the heat shock response or Hsp90 client degradation. The most efficacious molecules, **26l** and **37b**, were examined for their physiochemical profiles. Compound **37b** exhibits an improved physiochemical profile and therefore represent potent lead compounds for subsequent *in vivo* use.

## 4. Materials and methods

### 4.1. Chemistry

All reagents and solvents were purchased from commercial sources. ^1^H and ^13^C NMR spectra were recorded with a Bruker 400. Chemical shifts are referenced to the residual solvent peak and signals are quoted as δ values in parts per million (ppm). Coupling constants (J) are reported in Hertz. Flash column chromatography was performed using silica gel (irregular, 40-63 μm particle size, 60 Å pore size). Thin layer chromatography was performed with TLC silica gel 60F254 glass backed plates purchased from Millipore Sigma and visualized using UV light (254 nm). High resolution mass spectral data were obtained on a time-of-flight mass spectrometer (Brunker micrOTOF II), and analysis was performed using electrospray ionization. Data were acquired by the M1 method with positive ionization and set capillary 4500 V. Mass was reported as *m/z* scanning from 50 to 1650 *m/z*. HPLC purity analysis was determined using an Agilent 1260 Infinity II HPLC system with an autosampler (Agilent) eluting a C-18 reverse-phase column (Agilent Eclipse XDB-C18, 5 μm, 9.4 mm, x 250 mm) with a gradient mobile phase flow (4.0 mL/min; 0-100% acetonitrile with water for 20 min). Samples were monitored under UV light at 254 nm, and all tested compounds are ≥95% pure by HPLC analysis (major area peak/total combined area of peaks) unless otherwise stated.

#### 4.1.1. Synthesis of 3′,6-dimethoxy-[1,1′-biphenyl]-3-amine (3)

A 100 mL pressure vessel equipped with a magnetic stir bar was charged with 3-iodo-4-methoxyaniline (1.00 g, 4.015 mmol), (3-methoxyphenyl)boronic acid (0.793 g, 5.220 mmol) and potassium carbonate (1.67 g, 12.1 mmol) then dissolved in 1,4-dioxane (25 mL). The vessel was purged with argon then Pd(dppf)Cl_2_ (10 mol%) was added and the vessel was sealed for 16 h and stirred at 120 °*C* [15]. The mixture was cooled and filtered through a pad of celite with EtOAc. The filtrate was washed twice with deionized water and once with brine, then dried over Na_2_SO_4_ and concentrated *in vacuo*. The product was isolated by silica gel flash chromatography (1:3 EtOAc:Hexanes) 70.6% yield.

#### 4.1.2. Synthesis of 5-azido-2,3′-dimethoxy-1,1′-biphenyl (4)

A 250 mL round bottomed flask equipped with a magnetic stir bar was charged with 3′,6-dimethoxy-[1,1′-biphenyl]-3-amine (650 mg, 2.84 mmol) and dissolved in diluted sulfuric acid (5 mL, 1:7 sulfuric acid:water). This stirred at 0 °*C* and aqueous sodium nitrite (586 mg, 8.50 mmol) was added dropwise and allowed to stir for 30 min. Then aqueous sodium azide (737 mg, 11.3 mmol) was added dropwise and stirred for 1 h. At this time, the mixture was warmed to 25 °*C* to stir for 16 h [9]. The reaction was diluted with water and hexanes. The water layer was extracted 3 times with hexanes, then the organic layer was dried over sodium sulfate and concentrated *in vacuo*. The product was isolated by silica gel flash chromatography (1:5 EtOAc:Hexanes) 65% yield.

#### 4.1.3. Synthesis of compounds 6a-f

A 10 mL pressure vessel equipped with a magnetic stir bar was charged with 5-azido-2,3′-dimethoxy-1,1′-biphenyl (40 mg, 0.11 mmol) and dissolved in 1,4-dioxane (1.0 mL). Then a substituted ethynyl benzene (19.0 mg, 0.11 mmol) was added and the vessel was briefly purged with argon. Then, C_p_Ru(PPh_3_)_2_Cl or C_p_Ru(COD)Cl was added (10 mol%) and sealed by crimp cap. The reaction proceeded for 16 h at 80 °*C*, after which the mixture was cooled and filtered through a pad of celite with EtOAc [10,11]. The filtrate was washed twice with deionized water and once with brine, then dried over Na_2_SO_4_ and concentrated *in vacuo*. 20-45% yield. Products were isolated by silica gel flash chromatography.

#### 4.1.4. Synthesis of methyl 4-(3-methoxyphenyl)picolinate (8)

A 20 mL pressure vessel equipped with a magnetic stir bar was charged with methyl 4-bromopicolinate (0.500 g, 2.31 mmol), (3-methoxyphenyl)boronic acid (0.528 g, 3.47 mmol) and potassium carbonate (0.960 g, 6.94 mmol) then dissolved in 1,4-dioxane (10.0 mL). The vessel was purged with argon then Pd(dppf)Cl_2_ (10 mol%) was added and the vessel was sealed for 16 h and stirred at 120 °*C*. The mixture was cooled and filtered through a pad of celite with EtOAc. The filtrate was washed twice with deionized water and once with brine, then dried over Na_2_SO_4_ and concentrated *in vacuo*. The product was isolated by silica gel flash chromatography (1:3 EtOAc:Hex) 42.5% yield.

#### 4.1.5. Synthesis of 4-(3-methoxyphenyl)picolinic acid (9)

A 250 mL round bottomed flask with a magnetic stir bar was charged with methyl 4-(3-methoxyphenyl)picolinate (0.237 g, 0.975 mmol) and lithium hydroxide (0.117 g, 4.87 mmol) and dissolved in 3:1:1 THF: MeOH:water (13.0 mL). This mixture stirred for 16 h, then concentrated *in vacuo*. The mixture was acidified with 12 M HCl to pH 1-2 where a white precipitate formed. The aqueous layer was extracted with ethyl acetate 3 times, dried over sodium sulfate and concentrated *in vacuo* to yield pure product 80.4% yield.

#### 4.1.6. Synthesis of 4-(3-methoxyphenyl)picolinoyl chloride (10)

A dry 250 mL round bottomed flask with a dry magnetic stir bar was charged with 4-(3-methoxyphenyl)picolinic acid (0.200 g, 0.872 mmol) and dissolved in anhydrous DCM (3.0 mL) under argon atmosphere. The mixture was moved to 0 °*C* and catalytic DMF was added, then oxalyl chloride (0.335 g, 2.62 mmol, 0.236 mL) was added dropwise. The reaction was moved to 25 °*C* for 5 h. The mixture was concentrated *in vacuo* and carried forward.

#### 4.1.7. Synthesis of 4-(3-methoxyphenyl)-N-(4-(trifluoromethyl)phenyl) picolinamide (12)

A dry 250 mL round bottomed flask with a dry magnetic stir bar was charged with anhydrous DCM (3.0 mL), N-ethyl-N-isopropylpropan-2-amine (0.13 g, 1.0 mmol), and 4-(trifluoromethyl)aniline (0.065 g, 0.40 mmol) under argon atmosphere and stirred for 30 min at 25 °*C*. This mixture was moved to 0 °*C*. Then, 4-(3-methoxyphenyl)picolinoyl chloride (0.050 g, 0.20 mmol) was dissolved in anhydrous DCM (3.0 mL) and added dropwise to the reaction mixture. The reaction mixture was allowed to stir at 25 °*C* for 16 h, then washed 2 times with deionized H_2_O and once with brine. The organic layer was dried over sodium sulfate, extracted with DCM and concentrated *in vacuo*. The product was isolated by silica gel flash chromatography (1:2.5 EtOAc:Hex) 40% yield.

#### 4.1.8. Synthesis of 4-(3-methoxyphenyl)-2-(1-(4-(trifluoromethyl)phenyl)-1H-tetrazol-5-yl)pyridine (13)

A dry 10 mL pressure vial with a dry magnetic stir bar was charged with 4-(3-methoxyphenyl)-N-(4-(trifluoromethyl)phenyl)picolinamide (0.030g, 0.081 mmol) and dissolved in anhydrous ACN (0.8 mL). Then phosphorous(V) oxychloride (0.062 g, 0.40 mmol), and sodium azide (0.01 g, 0.16 mmol) were added. The reaction mixture was allowed to stir at 90 °*C* for 16 h. The reaction was cooled to 25 °*C*, neutralized with saturated aqueous sodium bicarbonate and extracted with EtOAc. The organic layer was washed 2 times with deionized H_2_O, once with brine, dried over sodium sulfate and concentrated *in vacuo*. The product was isolated by silica gel flash chromatography (1:5 EtOAc:Hex) 35% yield.

#### 4.1.9. Synthesis of 5-(3-methoxyphenyl)pyridin-3-amine (15a)

A 100 mL pressure vessel equipped with a magnetic stir bar was charged with 5-bromopyridin-3-amine (500 mg, 2.89 mmol), (3-methoxyphenyl)boronic acid (0.571 g, 3.76 mmol) and potassium carbonate (1.18 g, 8.52 mmol) then dissolved in 1,4-dioxane (10 mL). The vessel was purged with argon, then Pd(dppf)Cl_2_ (10 mol%) was added and the vessel was sealed for 16 h and stirred at 120 °*C*. The mixture was cooled to 25 °*C* and filtered through a pad of celite with EtOAc. The filtrate was washed twice with deionized water and once with brine, then dried over Na_2_SO_4_ and concentrated *in vacuo*. The product was isolated by silica gel flash chromatography (1:5 EtOAc:Hexanes) 55% yield.

#### 4.1.10. Synthesis of 3-azido-5-(3-methoxyphenyl)pyridine (16a)

A 250 mL round bottomed flask equipped with a magnetic stir bar was charged with 5-(3-methoxyphenyl)pyridin-3-amine (200 mg, 1.00 mmol) and dissolved in diluted sulfuric acid (1.5 mL, 1:7 sulfuric acid:water). This stirred at 0 °*C* and aqueous sodium nitrite (207 mg, 3.00 mmol) was added dropwise and allowed to stir for 30 min. Then aqueous sodium azide (260 mg, 4.00 mmol) was added dropwise and stirred for 1 h. At this time, the mixture was warmed to 25 °*C* to stir for 16 h. The reaction was diluted with an aqueous solution of saturated sodium bicarbonate and hexanes. The aqueous layer was extracted 3 times with hexanes, then the organic layer was dried over sodium sulfate and concentrated *in vacuo*. 60% yield.

#### 4.1.11. Synthesis of 3-(3-methoxyphenyl)-5-(5-(4-(trifluoromethyl) phenyl)-1H-1,2,3-triazol-1-yl)pyridine (18a)

A 10 mL pressure vessel equipped with a magnetic stir bar was charged with 3-azido-5-(3-methoxyphenyl)pyridine (75 mg, 0.33 mmol) and dissolved in 1,4-dioxane (1.5 mL). Then 1-ethynyl-4-(trifluoromethyl)benzene (56.0 mg, 0.33 mmol) was added and the vessel was briefly purged with argon. Then, C_p_Ru(PPh_3_)_2_Cl was added (10 mol%) and sealed by crimp cap. The reaction proceeded for 16 h at 80 °*C*, after which the mixture was cooled and filtered through a pad of celite with EtOAc. The filtrate was washed twice with deionized water and once with brine, then dried over Na_2_SO_4_ and concentrated *in vacuo*. The product was isolated by silica gel flash chromatography (1:5 EtOAc: Hexanes) 35% yield.

#### 4.1.12. Synthesis of 4-chloro-3′-methoxy-[1,1′-biphenyl]-3-amine (15b)

A 20 mL pressure vessel equipped with a magnetic stir bar was charged with 5-bromo-2-chloroaniline (500 mg, 2.89 mmol), (3-methoxyphenyl)boronic acid (0.552g, 3.63 mmol) and potassium carbonate (1.00 g, 8.52 mmol) then dissolved in 1,4-dioxane (10 mL). The vessel was purged with argon, then Pd(dppf)Cl_2_ (10 mol%) was added and the vessel was sealed for 16 h and stirred at 120 °*C*. The mixture was cooled to 25 °*C* and filtered through a pad of celite with EtOAc. The filtrate was washed twice with deionized water and once with brine, then dried over Na_2_SO_4_ and concentrated *in vacuo*. The product was isolated by silica gel flash chromatography (5% MeOH:DCM) 65% yield.

#### 4.1.13. Synthesis of 5-chloro-3′-methoxy-[1,1′-biphenyl]-3-amine (15c)

A 20 mL pressure vessel equipped with a magnetic stir bar was charged with 3-bromo-5-chloroaniline (500 mg, 2.89 mmol), (3-methoxyphenyl)boronic acid (0.552g, 3.63 mmol) and potassium carbonate (1.00 g, 8.52 mmol) then dissolved in 1,4-dioxane (10 mL). The vessel was purged with argon, then Pd(dppf)Cl_2_ (10 mol%) was added and the vessel was sealed for 16 h and stirred at 120 °*C*. The mixture was cooled to 25 °*C* and filtered through a pad of celite with EtOAc. The filtrate was washed twice with deionized water and once with brine, then dried over Na_2_SO_4_ and concentrated *in vacuo*. The product was isolated by silica gel flash chromatography (5% MeOH:DCM) 65.1% yield.

#### 4.1.14. Synthesis of 6-chloro-3′-methoxy-[1,1′-biphenyl]-3-amine (15d)

A 20 mL pressure vessel equipped with a magnetic stir bar was charged with 4-chloro-3-iodoaniline (500 mg, 1.97 mmol), (3-methoxyphenyl)boronic acid (450 mg, 2.96 mmol) and potassium carbonate (818 mg, 5.92 mmol) then dissolved in 1,4-dioxane (10 mL). The vessel was purged with argon, then Pd(dppf)Cl_2_ (10 mol%) was added and the vessel was sealed for 16 h and stirred at 120 °*C*. The mixture was cooled to 25 °*C* and filtered through a pad of celite with EtOAc. The filtrate was washed twice with deionized water and once with brine, then dried over Na_2_SO_4_ and concentrated *in vacuo*. The product was isolated by silica gel flash chromatography (5% MeOH:DCM) 65.1% yield.

#### 4.1.15. Synthesis of 3-azido-4-chloro-3′-methoxy-1,1′-biphenyl (16b)

A 250 mL round bottomed flask equipped with a magnetic stir bar was charged with 4-chloro-3′-methoxy-[1,1′-biphenyl]-3-amine (0.360 g, 1.54 mmol) and dissolved in diluted sulfuric acid (3.5 mL, 1:7 sulfuric acid:water). This stirred at 0 °*C* and aqueous sodium nitrite (0.319 g, 4.62 mmol) was added dropwise and allowed to stir for 30 min. Then aqueous sodium azide (0.401 g, 6.16 mmol) was added dropwise and stirred for 1 h. At this time, the mixture was warmed to 25 °*C* to stir for 16 h. The reaction was diluted with water and hexanes. The water layer was extracted 3 times with hexanes, then the organic layer was dried over sodium sulfate and concentrated *in vacuo*. The product was isolated by silica gel flash chromatography (1:5 EtOAc:Hexanes) 73.3% yield.

#### 4.1.16. Synthesis of 3-azido-5-chloro-3′-methoxy-1,1′-biphenyl (16c)

A 250 mL round bottomed flask equipped with a magnetic stir bar was charged with 5-chloro-3′-methoxy-[1,1′-biphenyl]-3-amine (0.300 g, 1.28 mmol) and dissolved in diluted sulfuric acid (3.5 mL, 1:7 sulfuric acid:water). This stirred at 0 °*C* and aqueous sodium nitrite (0.266 g, 3.85 mmol) was added dropwise and allowed to stir for 30 min. Then aqueous sodium azide (0.334 g, 5.13 mmol) was added dropwise and stirred for 1 h. At this time, the mixture was warmed to 25 °*C* to stir for 16 h. The reaction was diluted with water and hexanes. The water layer was extracted 3 times with hexanes, then the organic layer was dried over sodium sulfate and concentrated *in vacuo*. The product was isolated by silica gel flash chromatography (1:5 EtOAc:Hexanes) 67.8% yield.

#### 4.1.17. Synthesis of 5-azido-2-chloro-3′-methoxy-1,1′-biphenyl (16d)

A 250 mL round bottomed flask equipped with a magnetic stir bar was charged with 6-chloro-3′-methoxy-[1,1′-biphenyl]-3-amine (0.300 g, 1.28 mmol) and dissolved in diluted sulfuric acid (3.5 mL, 1:7 sulfuric acid:water). This stirred at 0 °*C* and aqueous sodium nitrite (0.266 g, 3.85 mmol) was added dropwise and allowed to stir for 30 min. Then aqueous sodium azide (0.335 g, 5.13 mmol) was added dropwise and stirred for 1 h. At this time, the mixture was warmed to 25 °*C* to stir for 16 h. The reaction was diluted with water and hexanes. The water layer was extracted 3 times with hexanes, then the organic layer was dried over sodium sulfate and concentrated *in vacuo*. The product was isolated by silica gel flash chromatography (1:5 EtOAc:Hexanes) 41.7% yield.

#### 4.1.18. Synthesis of 1-(4-chloro-3′-methoxy-[1,1′-biphenyl]-3-yl)-5-(4-(trifluoromethyl)phenyl)-1H-1,2,3-triazole (18b)

A 10 mL pressure vessel equipped with a magnetic stir bar was charged with 3-azido-4-chloro-3′-methoxy-1,1′-biphenyl (0.060 g, 0.23 mmol) and dissolved in 1,4-dioxane (2.0 mL). Then 1-ethynyl-4-(trifluoromethyl)benzene (0.049 g, 0.29 mmol) was added and the vessel was briefly purged with argon. Then, C_p_Ru(PPh_3_)_2_Cl was added (10 mol%) and sealed by crimp cap. The reaction proceeded for 16 h at 80 °*C*, after which the mixture was cooled to 25 °*C* and filtered through a pad of celite with EtOAc. The filtrate was washed twice with deionized water and once with brine, then dried over Na_2_SO_4_ and concentrated *in vacuo*. The product was isolated by silica gel flash chromatography (1:4 EtOAc:Hexanes). 55% yield.

#### 4.1.19. Synthesis of 1-(5-chloro-3′-methoxy-[1,1′-biphenyl]-3-yl)-5-(4-(trifluoromethyl)phenyl)-1H-1,2,3-triazole (18c)

A 10 mL pressure vessel equipped with a magnetic stir bar was charged with 3-azido-5-chloro-3′-methoxy-1,1′-biphenyl (60 mg, 0.23 mmol) and dissolved in benzene (1.7 mL). Then 1-ethynyl-4-(trifluoromethyl)benzene (0.039 mg, 0.23 mmol) was added and the vessel was briefly purged with argon. Then, C_p_Ru(COD)_2_Cl was added (10 mol%) and sealed by crimp cap. The reaction proceeded for 72 h at 80 °*C*, after which the mixture was cooled to 25 °*C* and filtered through a pad of celite with EtOAc. The filtrate was washed twice with deionized water and once with brine, then dried over Na_2_SO_4_ and concentrated *in vacuo*. The product was isolated by silica gel flash chromatography (1:4 EtOAc:Hexanes). 55% yield.

#### 4.1.20. Synthesis of 1-(6-chloro-3′-methoxy-[1,1′-biphenyl]-3-yl)-5-(4-(trifluoromethyl)phenyl)-1H-1,2,3-triazole (18d)

A 10 mL pressure vessel equipped with a magnetic stir bar was charged with 5-azido-2-chloro-3′-methoxy-1,1′-biphenyl (0.060 mg, 0.23 mmol) and dissolved in 1,4-dioxane (2.0 mL). Then 1-ethynyl-4-(trifluoromethyl)benzene (0.049 mg, 0.29 mmol) was added and the vessel was briefly purged with argon. Then, C_p_Ru(PPh_3_)_2_Cl was added (10 mol%) and sealed by crimp cap. The reaction proceeded for 16 h at 80 °*C*, after which the mixture was cooled to 25 °*C* and filtered through a pad of celite with EtOAc. The filtrate was washed twice with deionized water and once with brine, then dried over Na_2_SO_4_ and concentrated *in vacuo*. The product was isolated by silica gel flash chromatography (1:4 EtOAc:Hexanes). 67.1% yield.

#### 4.1.21. Synthesis of 4-azido-2-bromopyridine (20)

A 250 mL round bottomed flask equipped with a magnetic stir bar was charged with 2-bromopyridin-4-amine (670 mg, 3.87 mmol) and dissolved in diluted sulfuric acid (5 mL, 1:7 sulfuric acid:water). The mixture stirred at 0 °*C* and aqueous sodium nitrite (802 mg, 11.60 mmol) was added dropwise and allowed to stir for 30 min. Then aqueous sodium azide (1.01 g, 15.5 mmol) was added dropwise and stirred for 1 h. At this time, the mixture was warmed to 25 °*C* to stir for 16 h. The reaction was diluted with saturated aqueous sodium bicarbonate and hexanes. The water layer was extracted 3 times with hexanes, then the organic layer was dried over sodium sulfate and concentrated *in vacuo*. The product was isolated by silica gel flash chromatography (3% MeOH:DCM) 73% yield.

#### 4.1.22. Synthesis of 2-bromo-4-(5-(4-(trifluoromethyl)phenyl)-1H-1,2,3-triazol-1-yl)pyridine (21)

A 10 mL pressure vessel equipped with a magnetic stir bar was charged with 4-azido-2-bromopyridine (100 mg, 0.50 mmol) and dissolved in 1,4-dioxane (1.0 mL). Then 1-ethynyl-4-(trifluoromethyl)benzene (85.5 mg, 0.50 mmol) was added and the vessel was briefly purged with argon. Then, C_p_Ru(PPh_3_)_2_Cl was added (10 mol%) and sealed by crimp cap. The reaction proceeded for 4 h at 100 °*C*, after which the mixture was cooled and diluted with EtOAc. The filtrate was washed twice with deionized water and once with brine, then dried over Na_2_SO_4_ and concentrated *in vacuo*. The product was isolated by silica flash chromatography (4% MeOH:DCM). 25% yield.

#### 4.1.23. Synthesis of 2-(3-methoxyphenyl)-4-(5-(4-(trifluoromethyl) phenyl)-1H-1,2,3-triazol-1-yl)pyridine (22)

A 10 mL pressure vessel equipped with a magnetic stir bar was charged with 2-bromo-4-(5-(4-(trifluoromethyl)phenyl)-1H-1,2,3-triazol-1-yl)pyridine (0.05 g, 0.14 mmol), (3-methoxyphenyl)boronic acid (0.03 g, 0.2 mmol) and potassium carbonate (0.094 g, 0.68 mmol) then dissolved in 1,4-dioxane (1.0 mL). The vessel was purged with argon then Pd(dppf)Cl_2_ (10 mol%) was added and the vessel was sealed for 16 h and stirred at 120 °*C*. The mixture was cooled and filtered through a pad of celite with EtOAc. The filtrate was washed twice with deionized water and once with brine, then dried over Na_2_SO_4_ and concentrated *in vacuo*. The product was isolated by silica gel flash chromatography (1% MeOH:DCM) 20.2% yield.

#### 4.1.24. Synthesis of 4-azido-2-iodo-1-methoxybenzene (23)

A 250 mL round bottomed flask equipped with a magnetic stir bar was charged with 3-iodo-4-methoxyaniline (2.00 g, 8.03 mmol) and dissolved in diluted sulfuric acid (14 mL, 1:7 sulfuric acid:water). The mixture moved to 0 °*C* and aqueous sodium nitrite (1.66 g, 24.1 mmol) was added dropwise and allowed to stir for 30 min. Then aqueous sodium azide (2.09 g, 32.1 mmol) was added dropwise and stirred for 1 h. At this time, the mixture was warmed to 25 °*C* to stir for 16 h. The reaction was diluted with water and hexanes. The water layer was extracted 3 times with hexanes, then the organic layer was dried over sodium sulfate and concentrated *in vacuo*. The product was isolated by silica gel flash chromatography (1:10 EtOAc:Hexanes) 85% yield.

#### 4.1.25. Synthesis of 1-(3-iodo-4-methoxyphenyl)-5-(4-(trifluoromethyl) phenyl)-1H-1,2,3-triazole (24)

A 20 mL pressure vessel equipped with a magnetic stir bar was charged with 4-azido-2-iodo-1-methoxybenzene (1.00 g, 3.64 mmol) and dissolved in toluene (10 mL). Then 1-ethynyl-4-(trifluoromethyl) benzene (0.619 g, 3.64 mmol) was added and the vessel was briefly purged with argon. Then, C_p_Ru(PPh_3_)_2_Cl was added (10 mol%) and sealed by crimp cap. The reaction proceeded for 4 h at 80 °*C*, after which the mixture was cooled and diluted with EtOAc. This was washed twice with deionized water and once with brine, then dried over Na_2_SO_4_ and concentrated *in vacuo*. The product was isolated by silica flash chromatography (1:3 EtOAc:Hex). 22% yield.

#### 4.1.26. Synthesis of compounds 26a-g

A 10 mL pressure vessel equipped with a magnetic stir bar was charged with 1-(3-iodo-4-methoxyphenyl)-5-(4-(trifluoromethyl) phenyl)-1H-1,2,3-triazole (0.025 g, 0.056 mmol), substituted phenyl boronic acids/phenylboronic acid pinacol esters (0.11 mmol) and potassium carbonate (0.03 g, 0.2 mmol) then dissolved in 1,4-dioxane or DMF (0.5 mL). The vessel was purged with argon then Pd(dppf)Cl_2_, Pd (PPh_3_)_2_Cl_2_ or RuPhos PdG4(10 mol%) was added and the vessel was sealed for 16 h and stirred at 120 °*C*. The mixture was cooled and filtered through a pad of celite with EtOAc. Filtrate was washed twice with deionized water and once with brine, then dried over Na_2_SO_4_ and concentrated *in vacuo*. The product was isolated by silica gel flash chromatography 10-40% yield.

#### 4.1.27. Synthesis of compounds 26h-n

A 10 mL pressure vessel equipped with a magnetic stir bar was charged with 1-(3-iodo-4-methoxyphenyl)-5-(4-(trifluoromethyl) phenyl)-1H-1,2,3-triazole (0.025 g, 0.056 mmol), various substituted phenylboronic acid pinacol esters (0.11 mmol) and potassium carbonate (0.03 g, 0.2 mmol) then dissolved in 1,4-dioxane or DMF (0.5 mL). The vessel was purged with argon then Pd(PPh_3_)_2_Cl_2_ (10 mol%) was added and the vessel was sealed for 16 h and stirred at 120 °*C*. The mixture was cooled and filtered through a pad of celite with EtOAc. Filtrate was washed twice with deionized water and once with brine, then dried over Na_2_SO_4_ and concentrated *in vacuo*. The product was isolated by silica gel flash chromatography 10-40% yield.

#### 4.1.28. Synthesis of 1-(4-methoxy-3-(tetrahydro-2H-pyran-4-yl)phenyl)-5-(4-(trifluoromethyl)phenyl)-1H-1,2,3-triazole (27)

A 10 mL round bottomed flask equipped with a magnetic stir bar was charged with 1-(3-(3,6-dihydro-2H-pyran-4-yl)-4-methoxyphenyl)-5-(4-(trifluoromethyl)phenyl)-1H-1,2,3-triazole (0.020 g, 0.005 mmol) and dissolved in 10:1 methanol:water (0.55 mL). The flask was purged with argon, then purged with H_2_. Then, Pd/C (0.053 g, 0.50 mmol) was added and the reaction stirred under H_2_ atmosphere (1 atm) for 16 h at 25 °*C*. At this time, the mixture was filtered through a pad of celite with EtOAc. The filtrate was dried over Na_2_SO_4_ and concentrated *in vacuo*. The product was isolated by silica (SiO_2_) flash column chromatography (1:3 EtOAc:Hexanes). Yield 20%.

#### 4.1.29. Synthesis of 1-(4-methoxy-3-(tetrahydrofuran-3-yl)phenyl)-5-(4-(trifluoromethyl)phenyl)-1H-1,2,3-triazole (28)

A 10 mL round bottomed flask equipped with a magnetic stir bar was charged with 1-(3-(2,5-dihydrofuran-3-yl)-4-methoxyphenyl)-5-(4-(trifluoromethyl)phenyl)-1H-1,2,3-triazole (0.005 g, 0.01 mmol) and dissolved in EtOAc (0.2 mL). The flask was purged with argon, then purged with H_2_. Then, Pd/C (0.01 g, 0.10 mmol) was added and the reaction stirred under H_2_ atmosphere (1 atm) for 16 h at 25 °*C*. At this time, the mixture was filtered through a pad of celite with EtOAc. The filtrate was dried over Na_2_SO_4_ and concentrated *in vacuo*. The product was isolated by silica (SiO_2_) flash column chromatography (1:3 EtOAc:Hexanes). Yield 40%.

#### 4.1.30. Synthesis of 4-(2-methoxy-5-(5-(4-(trifluoromethyl)phenyl)-1H-1,2,3-triazol-1-yl)phenyl)-1,2,3,6-tetrahydropyridine (29)

A 10 mL round bottomed flask equipped with a magnetic stir bar was charged with *tert*-butyl 4-(2-methoxy-5-(5-(4-(trifluoromethyl)phenyl)-1H-1,2,3-triazol-1-yl)phenyl)-3,6-dihydropyridine-1(2H)-carboxylate (0.050 g, 0.10 mmol) and dissolved in DCM. Then, 4 M HCl in dioxane (0.022 g, 0.60 mmol) was added dropwise. The reaction stirred at 25 °*C* for 16 h. The reaction was washed twice with deionized water and once with brine. The organic phase was dried over Na_2_SO_4_ and concentrated *in vacuo*. Yield 70%.

#### 4.1.31. Synthesis of 4-(2-methoxy-5-(5-(4-(trifluoromethyl)phenyl)-1H-1,2,3-triazol-1-yl)phenyl)-3,6-dihydropyridine-1(2H)-carbaldehyde (30)

A dry 10 mL pressure vessel equipped with a dry magnetic stir bar was charged with 4-(2-methoxy-5-(5-(4-(trifluoromethyl)phenyl)-1H-1,2,3-triazol-1-yl)phenyl)-1,2,3,6-tetrahydropyridine was dissolved in formic acid (0.5 mL). Then, ZnCl_2_ (0.001 g, 0.0072 mmol) was added and the reaction was stirred at 70 °*C* for 24 h. At this time the mixture was cooled to 25 °*C* and filtered through a pad of celite with EtOAc. The filtrate was washed twice with deionized water and once with brine. The organic phase was dried over Na_2_SO_4_ and concentrated *in vacuo*. The product was isolated by silica (SiO_2_) flash column chromatography (1:4 EtOAc:Hexanes). Yield 35%.

#### 4.1.32. Synthesis of 1-(4-(2-methoxy-5-(5-(4-(trifluoromethyl)phenyl)-1H-1,2,3-triazol-1-yl)phenyl)-3,6-dihydropyridin-1(2H)-yl)ethan-1-one (31)

A 100 mL round bottomed flask with a magnetic stir bar was charged with 4-(2-methoxy-5-(5-(4-(trifluoromethyl)phenyl)-1H-1,2,3-triazol-1-yl)phenyl)-1,2,3,6-tetrahydropyridine (0.050 g, 0.12 mmol) and dissolved in DCM (1.0 mL). Then, triethylamine (0.063 g, 0.62 mmol) was added and stirred for 20 min. At this time acetic anhydride (0.025 g, 0.25 mmol) was added and the reaction stirred at 25 °*C* for 16 h. The reaction was washed 3 times with 1 M aqueous HCl and dried over Na_2_SO_4_. The product was isolated by silica (SiO_2_) flash column chromatography (1:1 EtOAc:Hexanes). Yield 31%.

#### 4.1.33. Synthesis of 4-(2-methoxy-5-(5-(4-(trifluoromethyl)phenyl)-1H-1,2,3-triazol-1-yl)phenyl)morpholine (32)

A dry pressure vessel equipped with a dry magnetic stir bar was charged with 1-(3-iodo-4-methoxyphenyl)-5-(4-(trifluoromethyl) phenyl)-1H-1,2,3-triazole (0.02 g, 0.045 mmol) and dissolved in anhydrous DMF (0.5 mL). Then, potassium carbonate (0.02 g, 0.13 mmol), dimethylglycine (0.0005 g, 0.0045 mmol), copper(I) iodide (0.0051 g, 0.0027 mmol) and morpholine (0.039 g, 0.45 mmol) were added under argon atmosphere. The reaction was allowed to stir for 24 h at 120 °*C*. At this time the mixture was cooled to 25 °*C* and filtered through a pad of celite with EtOAc. The filtrate was washed twice with deionized water and once with brine. The organic phase was dried over Na_2_SO_4_ and concentrated *in vacuo*. The product was isolated by silica (SiO_2_) flash column chromatography (1:4 EtOAc:Hexanes). Yield 25%.

#### 4.1.34. Synthesis of 2-oxaspiro[3.5]non-6-en-7-yl trifluoromethanesulfonate (34)

A dry 50 mL round bottomed flask equipped with a dry magnetic stir bar was charged with 2-oxaspiro[3.5]nonan-7-one (0.100 g, 0.713 mmol) and dissolved in anhydrous THF (5.0 mL), then moved to −78 °*C*. Then, lithiumdiisoproylamide in THF (1.0 M, 0.084 g, 0.785 mmol) was added dropwise and stirred for 2 h [16]. At this time, 1,1,1-trifluoro-N-phenyl-N-((trifluoromethyl)sulfonyl)methanesulfonamide (0.306 g, 0.856 mmol) was added and the reaction was moved to 25 °*C* for 48 h. The reaction was diluted with EtOAc and was washed twice with deionized water and once with brine, then dried over Na_2_SO_4_ and concentrated *in vacuo*. The product was isolated by silica gel flash chromatography (1:4 EtOAc:Hex) 33% yield.

#### 4.1.35. Synthesis of 1-(4-methoxy-3-(4,4,5,5-tetramethyl-1,3,2-dioxaborolan-2-yl)phenyl)-5-(4-(trifluoromethyl)phenyl)-1H-1,2,3-triazole (35)

A dry 10 mL pressure vessel equipped with a dry magnetic stir bar was charged with 1-(3-iodo-4-methoxyphenyl)-5-(4-(trifluoromethyl) phenyl)-1H-1,2,3-triazole (0.200 g, 0.449 mmol) and 2-dicyclohexylphosphino-2,6-dimethoxy-1,1-biphenyl (0.015 g, 0.674 mmol), then purged with argon. Pd(CH_3_CN)_2_Cl_2_ (0.0023 g, 0.009 mmol) was added and dissolved in DMF (0.3 mL). Once dissolved, pinacolatoborane (0.086 g, 0.674 mmol) and diisopropylethylamine (0.290 g, 2.25 mmol) were added dropwise [13]. The reaction was moved to 80 °*C* and stirred for 24 h under argon atmosphere. The reaction was cooled to 25 °*C* and filtered through celite with EtOAc. The organic layer was washed twice with deionized H_2_O and once with brine then dried over Na_2_SO_4_ and concentrated *in vacuo*. The product was isolated by silica flash chromatography (1:4 EtOAc:Hex). 11.2% yield.

#### 4.1.36. Synthesis of 1-(4-methoxy-3-(2-oxaspiro[3.5]non-6-en-7-yl) phenyl)-5-(4-(trifluoromethyl)phenyl)-1H-1,2,3-triazole (37a)

A 10 mL pressure vessel equipped with a magnetic stir bar was charged with 1-(4-methoxy-3-(4,4,5,5-tetramethyl-1,3,2-dioxaborolan-2-yl)phenyl)-5-(4-(trifluoromethyl)phenyl)-1H-1,2,3-triazole (0.015 g, 0.034 mmol), 2-oxaspiro[3.5]non-6-en-7-yl trifluoromethanesulfonate (0.014 g, 0.051 mmol) and potassium carbonate (0.014 g, 0.010 mmol) then dissolved in 1,4-dioxane (0.5 mL). The vessel was purged with argon then Pd(PPh_3_)_2_Cl_2_ (10 mol%) was added and the vessel was sealed for 16 h and stirred at 120 °*C*. The mixture was cooled and filtered through a pad of celite with EtOAc. Filtrate was washed twice with deionized water and once with brine, then dried over Na_2_SO_4_ and concentrated *in vacuo*. The product was isolated by silica gel flash chromatography (1:4 EtOAc:Hex) 11% yield.

#### 4.1.37. Synthesis of 5-(2-methoxy-5-(5-(4-(trifluoromethyl)phenyl)-1H-1,2,3-triazol-1-yl)phenyl)pyrimidine (37b)

A 10 mL pressure vessel equipped with a magnetic stir bar was charged with 1-(4-methoxy-3-(4,4,5,5-tetramethyl-1,3,2-dioxaborolan-2-yl)phenyl)-5-(4-(trifluoromethyl)phenyl)-1H-1,2,3-triazole (0.025 g, 0.056 mmol), 5-bromopyrimidine (0.012 g, 0.073 mmol) and potassium carbonate (0.023 g, 0.17 mmol) then dissolved in 1,4-dioxane (0.5 mL). The vessel was purged with argon then Pd(dppf)Cl_2_ (10 mol%) was added and the vessel was sealed for 16 h and stirred at 120 °*C*. The mixture was cooled and filtered through a pad of celite with EtOAc. Filtrate was washed twice with deionized water and once with brine, then dried over Na_2_SO_4_ and concentrated *in vacuo*. The product was isolated by silica gel flash chromatography 35% yield.

#### 4.1.38. Synthesis of triisopropyl((4-(4,4,5,5-tetramethyl-1,3,2-dioxaborolan-2-yl)cyclohex-3-en-1-yl)oxy)silane (39)

A dry 10 mL round bottomed flask equipped with a dry magnetic stir bar was charged with 4-(4,4,5,5-tetramethyl-1,3,2-dioxaborolan-2-yl) cyclohex-3-en-1-ol (0.025 g, 0.11 mmol) and dissolved in anhydrous DCM (0.4 mL). Then, N-ethyl-N-isopropylpropan-2-amine (0.072 g, 0.56 mmol) was added and stirred for 20 min. At this time triisopropylsilyl tifluoromethanesulfonate (0.051 g, 0.17 mmol) was added and was allowed to stir for 16 h at 25 °*C*. The reaction mixture was diluted with DCM, washed twice with deionized water and once with brine, then dried over Na_2_SO_4_ and concentrated *in vacuo*. The product was isolated by silica gel flash chromatography (1:4 EtOAc:Hex) 71% yield.

#### 4.1.39. Synthesis of 1-(6-methoxy-4'-((triisopropylsilyl)oxy)-2′,3′,4′,5′-tetrahydro-[1,1′-biphenyl]-3-yl)-5-(4-(trifluoromethyl)phenyl)-1H-1,2,3-triazole (40)

A 10 mL pressure vessel equipped with a magnetic stir bar was charged with 1-(3-iodo-4-methoxyphenyl)-5-(4-(trifluoromethyl) phenyl)-1H-1,2,3-triazole (0.045 g, 0.13 mmol), triisopropyl((4-(4,4,5,5-tetramethyl-1,3,2-dioxaborolan-2-yl)cyclohex-3-en-1-yl)oxy) silane (0.05 g, 0.13 mmol) and cesium carbonate (0.07 g, 0.51 mmol) then dissolved in 1,4-dioxane (0.8 mL). The vessel was purged with argon, then Pd RuPhosG_4_ (10 mol%) was added and the vessel was sealed for 16 h and stirred at 120 °*C*. The mixture was cooled and filtered through a pad of celite with EtOAc. Filtrate was washed twice with deionized water and once with brine, then dried over Na_2_SO_4_ and concentrated *in vacuo*. The product was isolated by silica gel flash chromatography (1:5 EtOAc:Hex) 31% yield.

#### 4.1.40. Synthesis of 2′-methoxy-5'-(5-(4-(trifluoromethyl)phenyl)-1H-1,2,3-triazol-1-yl)-2,3,4,5-tetrahydro-[1,1′-biphenyl]-4-ol (41)

A 10 mL round bottomed flask equipped with a magnetic stir bar was charged with 1-(6-methoxy-4'-((triisopropylsilyl)oxy)-2′,3′,4′,5′-tetrahydro-[1,1′-biphenyl]-3-yl)-5-(4-(trifluoromethyl)phenyl)-1H-1,2,3-triazole (0.018 mg, 0.031 mmol) and dissolved in THF (0.5 mL). Then tetrabutylammonium fluoride (1.0 M in THF, 0.010 g, 0.038 mmol) was added and allowed to stir for 30 min at 25 °*C*. At which time the reaction was diluted with EtOAc and washed twice with deionized water and once with brine, then dried over Na_2_SO_4_ and concentrated *in vacuo*. The product was isolated by silica gel flash chromatography (1% MeOH: DCM) 50% yield.

#### 4.1.41. Synthesis of 1-(6-methoxy-4'-(trifluoromethoxy)-2′,3′,4′,5′-tetrahydro-[1,1′-biphenyl]-3-yl)-5-(4-(trifluoromethyl)phenyl)-1H-1,2,3-triazole (42)

A dry 10 mL round bottomed equipped with a dry magnetic stir bar was charged with 2′-methoxy-5'-(5-(4-(trifluoromethyl)phenyl)-1H-1,2,3-triazol-1-yl)-2,3,4,5-tetrahydro-[1,1′-biphenyl]-4-ol (0.070 g, 0.17 mmol), 1-Trifluoromethyl-1,2-benziodoxol-3-(1H)-one (Togni reagent II) as a 40% mixture on celite (11 mg, 0.034 mmol) and Zinc di[bis (trifluoromethanesulfonyl)imide] (0.021 g, 0.034 mmol) and dissolved in anhydrous CHCl_3_ (2.0 mL) under an argon atmosphere [14]. The mixture was allowed to stir for 48 h and dried over Na_2_SO_4_ and extracted with EtOAc. The filtrate was concentrated *in vacuo*, then purified by silica gel flash chromatography (1:1 Hex:EtOAc) 74% yield.

#### 4.1.42. Synthesis of 3-(1-(3-iodo-4-methoxyphenyl)-1H-1,2,3-triazol-5-yl) pyridine (43)

A 20 mL pressure vessel equipped with a magnetic stir bar was charged with 4-azido-2-iodo-1-methoxybenzene (0.150 g, 0.658 mmol) and dissolved in toluene (2.5 mL). Then 3-ethynylpyridine (0.082 mg, 0.789 mmol) was added and the vessel was briefly purged with argon. Then, C_p_Ru(COD)Cl was added (10 mol%) and sealed by crimp cap. The reaction proceeded for 4 h at 80 °*C*, after which the mixture was cooled and filtered through a pad of celite with EtOAc. The filtrate was washed twice with deionized water and once with brine, then dried over Na_2_SO_4_ and concentrated *in vacuo*. The product was isolated by silica gel flash chromatography (3% MeOH:DCM) 25% yield.

#### 4.1.43. Synthesis of 3-(1-(3-(3,6-dihydro-2H-pyran-4-yl)-4-methoxyphenyl)-1H-1,2,3-triazol-5-yl)pyridine (44)

A 10 mL pressure vessel equipped with a magnetic stir bar was charged with 3-(1-(3-iodo-4-methoxyphenyl)-1H-1,2,3-triazol-5-yl)pyridine (0.025 g, 0.075 mmol), 2-(3,6-dihydro-2H-pyran-4-yl)-4,4,5,5-tetramethyl-1,3,2-dioxaborolane (0.032 g, 0.15 mmol) and potassium carbonate (0.031 g, 0.23 mmol) then dissolved in 1,4-dioxane (0.6 mL). The vessel was purged with argon, then Pd(PPh_3_)_2_Cl_2_ (10 mol%) was added and the vessel was sealed for 16 h and stirred at 120 °*C*. The mixture was cooled and filtered through a pad of celite with EtOAc. Filtrate was washed twice with deionized water and once with brine, then dried over Na_2_SO_4_ and concentrated *in vacuo*. The product was isolated by silica gel flash chromatography (1% MeOH:DCM) 31% yield.

### 4.2. Split-renilla luciferase assay for compound screening

The following assay was performed according to the reported protocol [17]. HeLa cells were seeded in 10 cm cell culture dishes (VWR) at 2 × 10^6^ cells in DMEM supplemented with 10% HI-FBS (Gibco), 100 U/mL of penicillin and 100 μg/mL streptomycin (VWR). They were cultured for 2 days at 37 °*C*, 5% CO_2_ and saturated humidity. The growth medium was switched to DMEM without the antibiotic, then cells were transfected via Transporter5 (KyforaBio) according to the manufacturer protocol with 15 μg of the C-terminal *Renilla* luciferase-Aha1 or the N-terminal *Renilla* Luciferase-Hsp90 *α* plasmid DNA prepared by PureYield Midi-Prep (Promega). The antibiotic free media was swapped for media containing antibiotics after at least 6 h. Cells were lysed and pelleted 48 h post-transfection. The NRL-Hsp90 *α* and CRL-Aha1 supernatants were diluted in Millipore water 1:17 in a master mix. 100 μL of this was added to each sample with 0.7% DMSO or drug. The samples incubated for 1 h at which time 30 μL of each sample were added to a white flat bottomed 96 well PS plate in triplicate, then 20 μL of coelentarezine from the *Renilla* luciferase assay system (Promega) were added and luminescence was read immediately using a BioTek Cytation5 plate reader.

### 4.3. Co-Immunoprecipitation

This procedure closely follows the recently published protocol [8]. SK-Br-3 or SH-SY5Y cells were seeded in 10 cm dishes at a density of 1.5 × 10^6^ cells, then cultured for 24 h in DMEM (Gibco) supplemented with 10% HI-FBS (Gibco) and 100 U/mL penicillin and 100 μg/mL streptomycin (VWR) at 37 °*C*, 5% CO_2_ and saturated humidity. At this time, media was aspirated, and cells were treated with 10 μM compound or equal volume of DMSO (vehicle) in media for 24 h. Then, the cells were washed twice with cold PBS (Fisher Bio Reagents) and lysed using RIPA lysis buffer (130 mM NaCl, 1% Triton X-100, 1 mM EDTA, 0.1% SDS, 10 mM Tris-Cl, pH 8.0 in water), containing 1:100 Phosphatase Inhibitor Cocktail 2 (Sigma-Aldrich), 1:100 Phosphatase Inhibitor Cocktail 3 (Sigma-Aldrich), 1:100 Protease Inhibitor Cocktail Set III, EDTA-free (CalBiochem^®^) and 10 μM Phenylmethylsulfonyl fluoride on ice. After a 10-min incubation on ice, the cell suspension was centrifuged at 10,000×*g* for 10 min at 4 °*C*. Supernatants were aspirated and transferred to new respective tubes, while a bicinchoninic acid (BCA) assay (Thermo Scientific) was performed to quantify protein concentration. Aliquots of 500 μg protein from each supernatant were transferred to respective tubes and diluted to 1 μg/μL with Millipore water (final volume 500 μL). 50 μL of 1 μg/μL input samples were set aside for later. 4 ng of Aha1 primary antibody (anti-mouse, C-12, Santa Cruz Biotechnology, SC16600Z) was added to each tube. These samples were placed on a rocker at 4 °*C* overnight to immunoprecipitate Aha1.

The following day, 500 μL of Pierce^™^ Protein G Agarose resin (Thermo Scientific) was centrifuged at 10×*g* for 5 min at 4 °*C* and subjected to six wash cycles. Each wash cycle consisted of aspirating the supernatant, addition of 900 μL of cold 1x DPBS at 10,000×*g* for 5 min at 4 °*C*. The supernatant was aspirated following the final wash and the beads were diluted with cold 1x DPBS to make a 1:1 beads:DPBS slurry. 50 μL of this slurry was added to each sample and rocked for 3 h at 4 °*C*. Following incubation, the samples were centrifuged at 10,000×*g* for 5 min at 4 °*C*. Samples were subject to 6 wash cycles, which consisted of addition of 600 μL of cold 1x RIPA buffer, centrifugation at 10×*g* for 5 min at 4 °*C*. Following the final wash, the supernatant was removed and 30 μL of 2x Laemlli buffer containing 355 mM 2-mercaptoethanol were added to the samples, then the samples were boiled at 95 °*C* for 5 min. Input samples were diluted 1:1 with the same Laemlli+ *β* ME and boiled for 5 min. Co-IP samples were centrifuged at 10,000×*g* for 5 min at 4°*C*. 25 μL of the Co-IP samples were loaded into the wells of the 10% Mini-POTEAN^®^ TGX Stain-Free^™^ Protein Gel (10 well, 50 μL well volume, Bio-Rad) and 20 μL of input samples were added to a separate gel and electrophoresed at 120 V for 90 min. Proteins were transferred to a PVDF membrane using the Trans-Blot Turbo System (1.3 A, 25 V, 10 min; Bio-Rad). Membranes were blocked in 7% skim milk at 25 °*C* for 1 h, then incubated in primary antibody solution containing 1:1000 dilutions of primary antibody in 0.3% w/v sodium azide in 7% skim milk. Primary antibodies used for visualization included Hsp90 9D2 (Enzo) to probe for co-immunoprecipitated Hsp90, as well as Aha1 SPC-183 (Stressmarq). The input blot was probed for Her2/ErbB2 29D8 (Cell Signaling), Cdk6 DCS83 (Cell Signaling), Aha1 C-12 (Santa Cruz Biotechnology), Hsp90 9D2 (Enzo), Hsp70/72C92F3A-5 (Enzo) and *β*-actin 8H10D10 (Cell Signaling). Membranes were washed three times with 1x Tris buffered saline, pH 7.4 with 0.1% Tween-20 (TBST) prior to incubation with 1:2000 diluted, appropriate species-matched secondary antibody. Secondary antibodies used in these experiments were Antirabbit IgG, HRP-linked antibody (1:2000, Cell Signaling) and Goat Anti-mouse Ig, Human ads HRP (1:2000, SouthernBiotech), and Goat Anti-Rat Ig, Mouse ads-HRP (1:2000, SouthernBiotech). Blots were visualized using a ChemiDoc Imaging System (Bio-Rad).

### 4.4. Evaluation of MDCK-MDR1 monolayer integrity with Lucifer Yellow

Lucifer yellow (LY) measurements were carried out with 60 μM LY in HBSS. The basolateral chamber received 500 μL of transport medium and the apical chambers received 200 μL 60 μM LY. This plate incubated at 37 °*C* with 5% CO_2_ at saturated humidity for 1 h. At the end of this incubation, the fluorescence intensity of basolateral medium of wells treated with LY were read with a Biotek Cytation 5 plate reader with a 485/20 nm excitation filter, a 530/25 nm emission filter and a 510 nm cutoff dichroic mirror.

### 4.5. MDCK-MDR1 permeability

MDCK-MDR1 cells were cultured in DMEM supplemented with 10% FBS (VWR) and 100 U/mL penicillin and 100 μg/mL streptomycin (Hyclone) at 37 °*C*, 5% CO_2_ and saturated humidity. The cells were seeded at a density of 1.25*10^5^ cells/well of 24 well plate cell culture inserts with polycarbonate 0.4 μm pore size (Thermo Scientific). Monolayers were allowed to form over 6 days, where media was changed every other day. Monolayer integrity was determined with 60 μM Lucifer Yellow in wells containing monolayers (Supplementary Fig. 1). 50 μM drug stock solutions were prepared in HBSS. Then, cells were treated in the apical (200 μL final volume) or basolateral (500 μL final volume) compartment in duplicate. The respective sides that were not treated received transfer medium without compound. The assay proceeded for 1 h at 37 °*C*, 5% CO_2_ and saturated humidity. Then, 100 μL were removed from the basolateral chamber for apical treated groups and vice versa. The 100 μL were diluted 1:1 with acetonitrile. The analytes from the basolateral chamber were injected in a 2.5:1 ratio with the analytes from the apical compartment. The analyte was separated with an Agilent 1260 Infinity II HPLC system with an autosampler (Agilent) eluting a C-18 reverse-phase column (Agilent Eclipse plus C18, 3.5 μm, 4.6 mm, x 100 mm) with a gradient mobile phase flow (4.0 mL/min; 0-100% acetonitrile with water for 20 min). Samples were monitored under UV light at 254 nm. The apparent permeability was calculated by the following equation Papp=(CF∕t)∕(SACi∗), where $C_{F}$ is the drug concentration (μg/mL) in the receiving chamber after 1 h, SA is the monolayer surface area (cm^2^), $C_{i}$ is the initial concentration (μg/mL) and t is the assay time (seconds). The apparent permeability of apical to basolateral and basolateral to apical transport was used to calculate the efflux ratio (ER) of the compounds by the following equation: $\text{ER}=P_{\text{app}\;B\text{-}>A}∕P_{\text{app}\;A\text{-}>B}$.

### 4.6. Aqueous solubility

Aqueous solubility in phosphate buffered saline pH 7.4 was determined by diluting 10 mM DMSO drug stock solutions to 250 μM with 190 μL PBS pH 7.4 in a clear non-sterile 0.22 *μ* m hydrophilic low protein binding durapore membrane filter plate (Millipore) according to instructions by Millipore. Final well volumes were 200 *μ* L with 5% DMSO and the samples incubated at 25°*C* with agitation overnight. At this time the filter plate was stacked on a clear polystyrene flat bottomed non-sterile 96 well microplate (Corning) and centrifuged. A standard curve of known concentrations was generated for each drug. These samples were separated by an Agilent 1260 Infinity II HPLC system with an autosampler (Agilent) eluting a C-18 reverse-phase column (Agilent Eclipse plus C18, 3.5 μm, 4.6 mm, x 100 mm) with a gradient mobile phase flow (4.0 mL/min; 0-100% acetonitrile with water for 20 min). Samples were monitored under UV light at 254 nm. The solubility was determined interpolating the sample AUC with the standard curve.

### 4.7. hLM metabolic stability

Drug half-life was determined by adding the compound and human liver microsomes to assay buffer (sterile PBS pH 7.4, 3 mM MgCl_2_, 1 mM NADPH) at a final concentration of 50 *μ* M drug and 0.5 mg/mL microsomes with a final volume of 550 μL. This was allowed to incubate with agitation at 37 °*C*. Various time points were collected from 0 to 1500 min by removing 50 μL aliquots and quenching with cold ACN (1:5). These samples were separated by an Agilent 1260 Infinity II HPLC system with an autosampler (Agilent) eluting a C-18 reverse-phase column (Agilent Eclipse plus C18, 3.5 μm, 4.6 mm, x 100 mm) with a gradient mobile phase flow (4.0 mL/min; 0-100% acetonitrile with water for 20 min). Samples were monitored under UV light at 254 nm. Compound half-life was calculated by interpolating the AUC that is 50% of that measured at time zero.

## Supplementary Material

Supplemental Data

## Figures and Tables

**Fig. 1.**
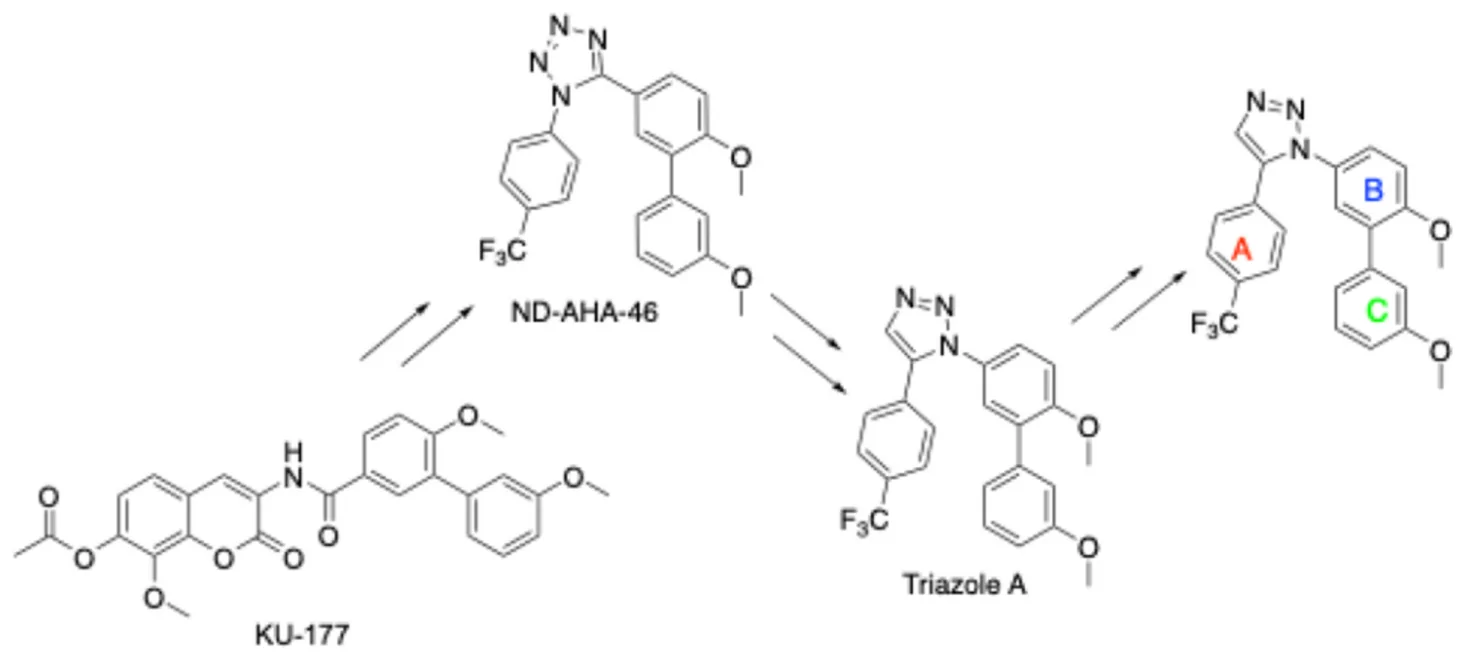
Parent lead compound KU-177 led to bioisosteric studies that revealed Triazole A as a new lead from tetrazole ND-AHA-46, where this work aims to derivatize rings A, B, and C to improve potency and druglike properties.

**Fig. 2.**
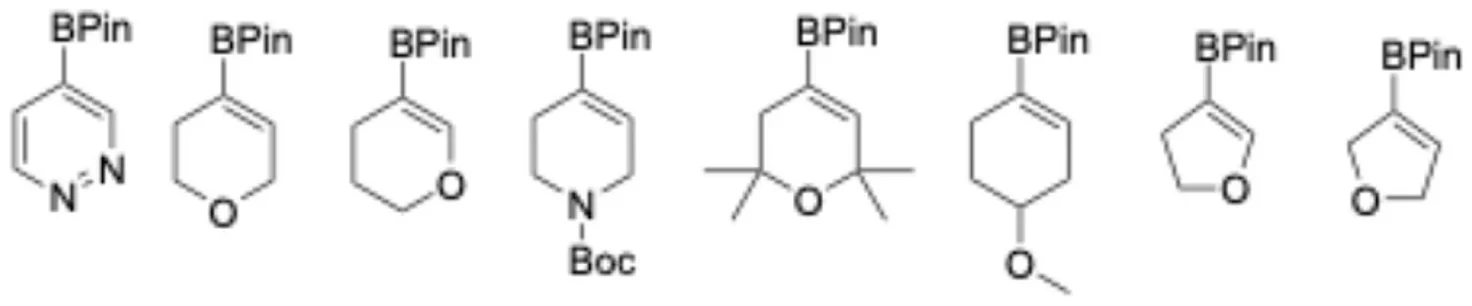
Products **26g-n** were prepared according to Scheme 5 with the boronic esters above. * Pd(PPh_3_)_2_Cl_2_.

**Fig. 3.**
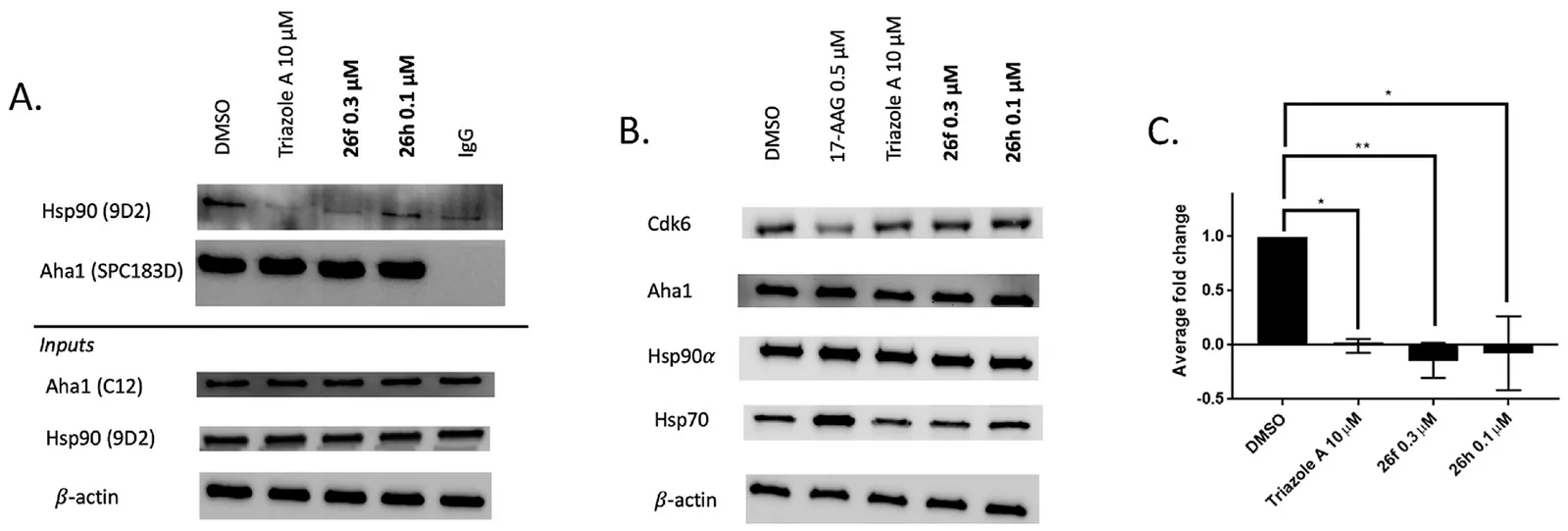
(A) SH-SY5Y cells were treated with compound for 24 h, then subject to Aha1 immunoprecipitation (IP), where Hsp90 *α* co-IP was evaluated by western blot. (B) SH-SY5Y cells were treated with DMSO, pan-inhibitor 17-AAG or Hsp90/Aha1 disruptor and client degradation and induction of the heat shock response was evaluated by western blot. (C) SK-Br-3 cells were treated with compound or DMSO for 24 h, then subject to Aha1 immunoprecipitation (IP), where Hsp90 *α* co-IP was evaluated by western blot (*n* = 3), *p < 0.05, **p < 0.005.

**Scheme 1.**
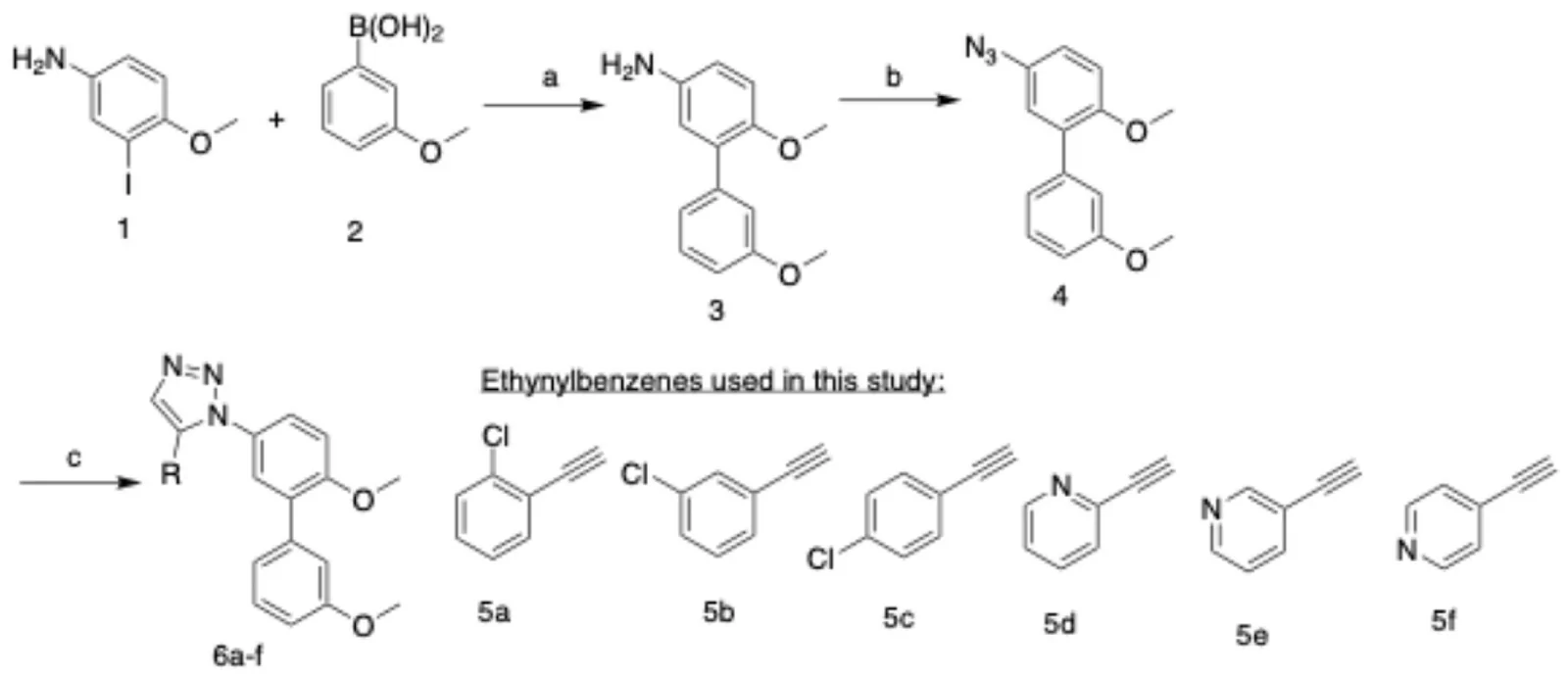
a) **1, 2**, K_2_CO_3_, Pd(dppf)Cl_2_, inert atmosphere, 1,4-dioxane, 120 °*C*,16 h 70.6%. b) **3** was added to dilute H_2_SO_4_ at 0 °*C*, then aqueous NaNO_2_ was added to stir for 1 h. Then aqueous NaN_3_ was added to stir for 1 h. The reaction was allowed to stir for 12 h at 25 °*C*, 65% and c) **4** and **5a-f** were dissolved in 1,4-dioxane, inert atmosphere, C_p_*RuCl(PPh_3_)_2_ or C_p_*Ru(COD)Cl (cat.), 80 °*C*, 16 h, 20-45% [9-11].

**Scheme 2.**
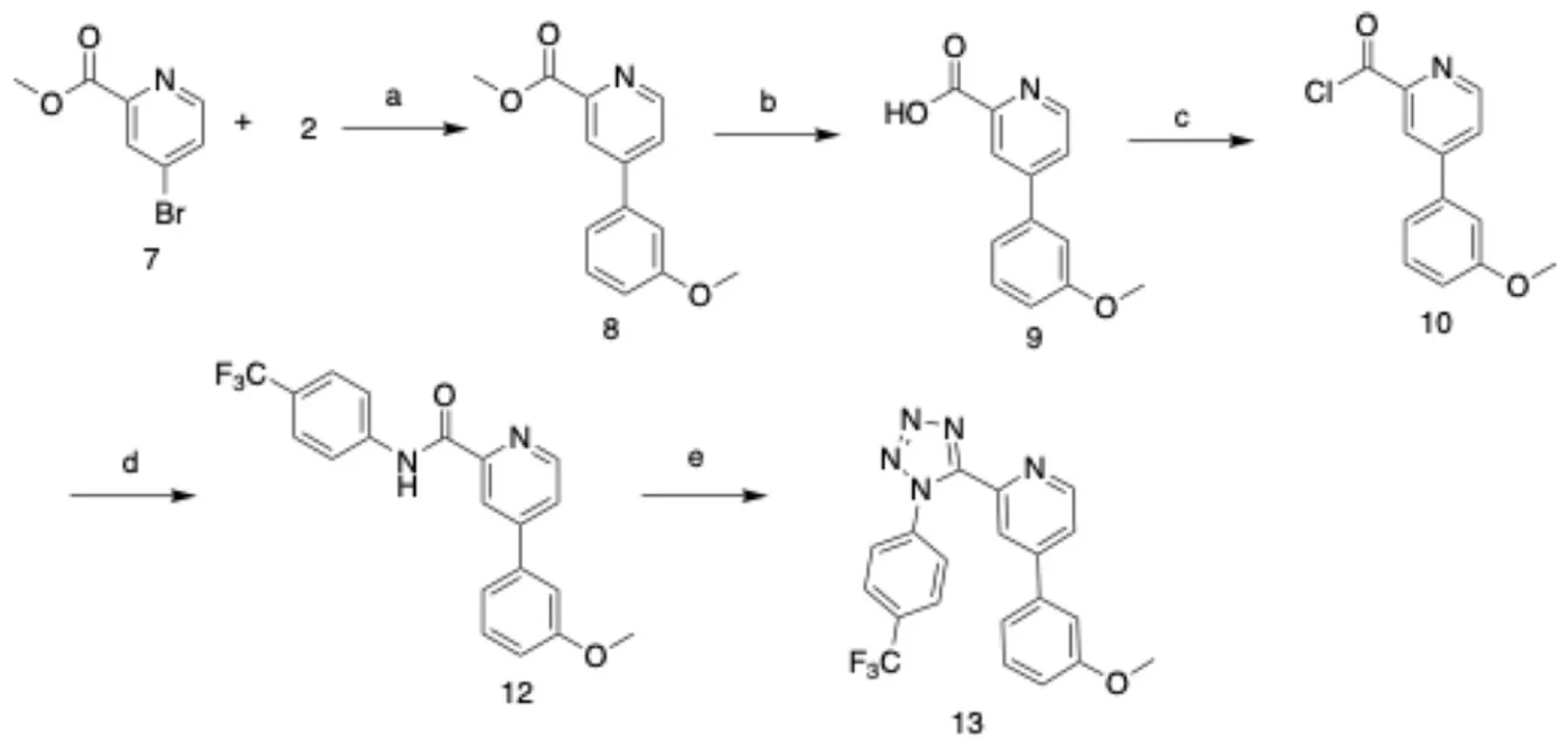
a) **7, 2**, K_2_CO_3_, Pd(dppf)Cl_2_, inert atmosphere, 1,4-dioxane, 120 °*C*,16 h 42.5%. b) **8**, LiOH, 3:1:1 THF:H_2_O:MeOH, 25 °*C*, 16 h 80.4%. c) **9**, DMF (cat.), oxalyl chloride, DCM, 25 °*C*, 5 h, carried forward. d) **10**, 4-(trifluoromethyl)aniline, DIPEA, 25 °*C*, 16 h 40%. e) **12**, POCl_3_, NaN_3_, ACN, 90 °*C*, 16 h 35% [13].

**Scheme 3.**
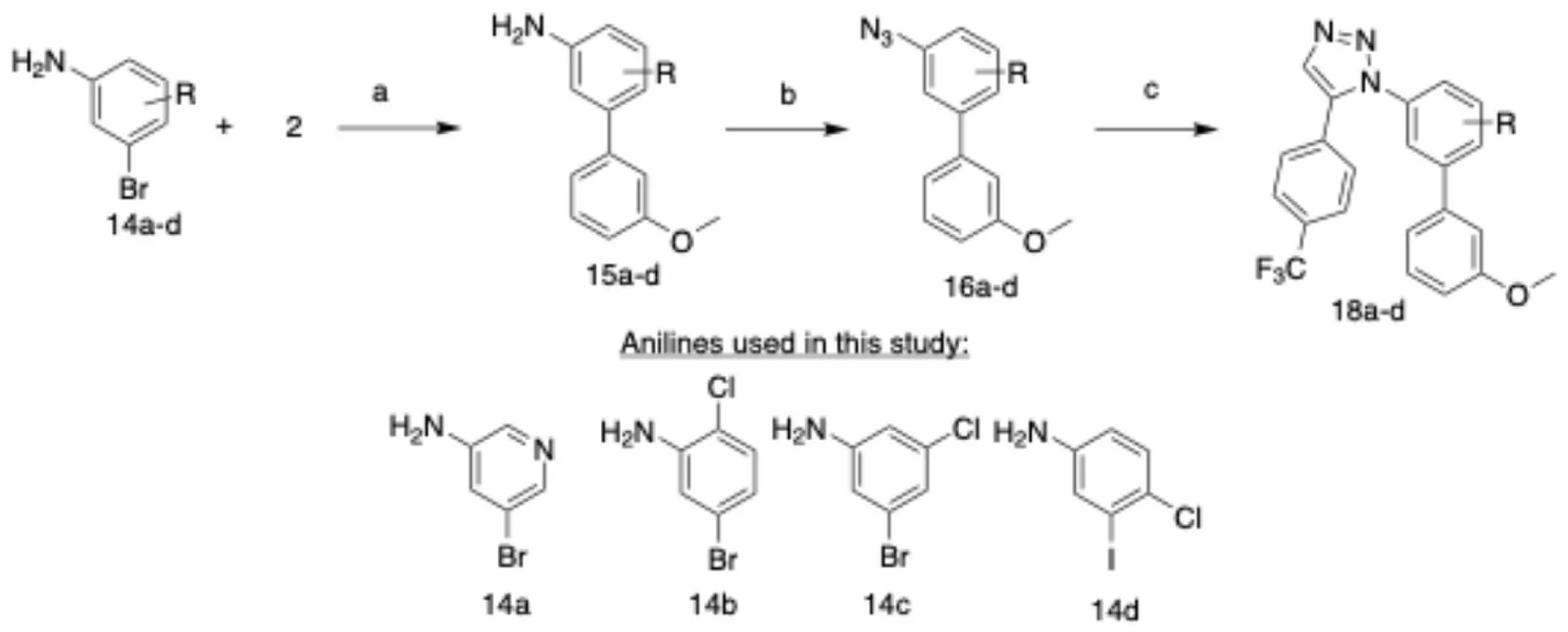
a) **14a-d, 2**, K_2_CO_3_, Pd(dppf)Cl_2_, inert atmosphere, 1,4-dioxane, 120 °*C*,16 h 40-65%. (b) **15a-d** was added to dilute H_2_SO_4_ at 0 °*C*, then aqueous NaNO_2_ was added to stir for 1 h. Then aqueous NaN_3_ was added to stir for 1 h. The reaction was allowed to stir for 12 h at 25 °*C*, 65-75%. (c) **16a-d** and 4-trifluoromethylethynylbenzene (**17**) were dissolved in 1,4-dioxane, inert atmosphere, C_p_*RuCl(PPh_3_)_2_ (cat.), 80 °*C*, 16 h, 35-67%.

**Scheme 4.**
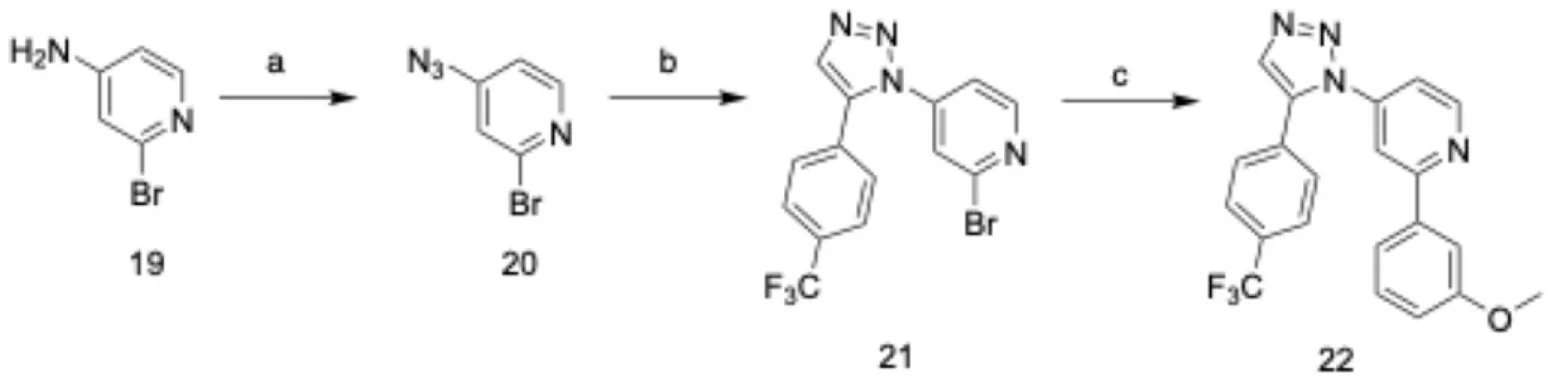
a) **19** was added to dilute H_2_SO_4_ at 0 °*C*, then aqueous NaNO_2_ was added to stir for 1 h. Then aqueous NaN_3_ was added to stir for 1 h. The reaction was allowed to stir for 12 h at 25 °*C*, 73%. b) **20** and **17** were dissolved in 1,4-dioxane, argon atmosphere, C_p_*RuCl(PPh_3_)_2_(cat.), 80 °*C*, 16 h, 25%. c) **21**, 2, K_2_CO_3_, Pd (dppf)Cl_2_, argon atmosphere, 1,4-dioxane, 120 °*C*,16 h 20.2%.

**Scheme 5.**
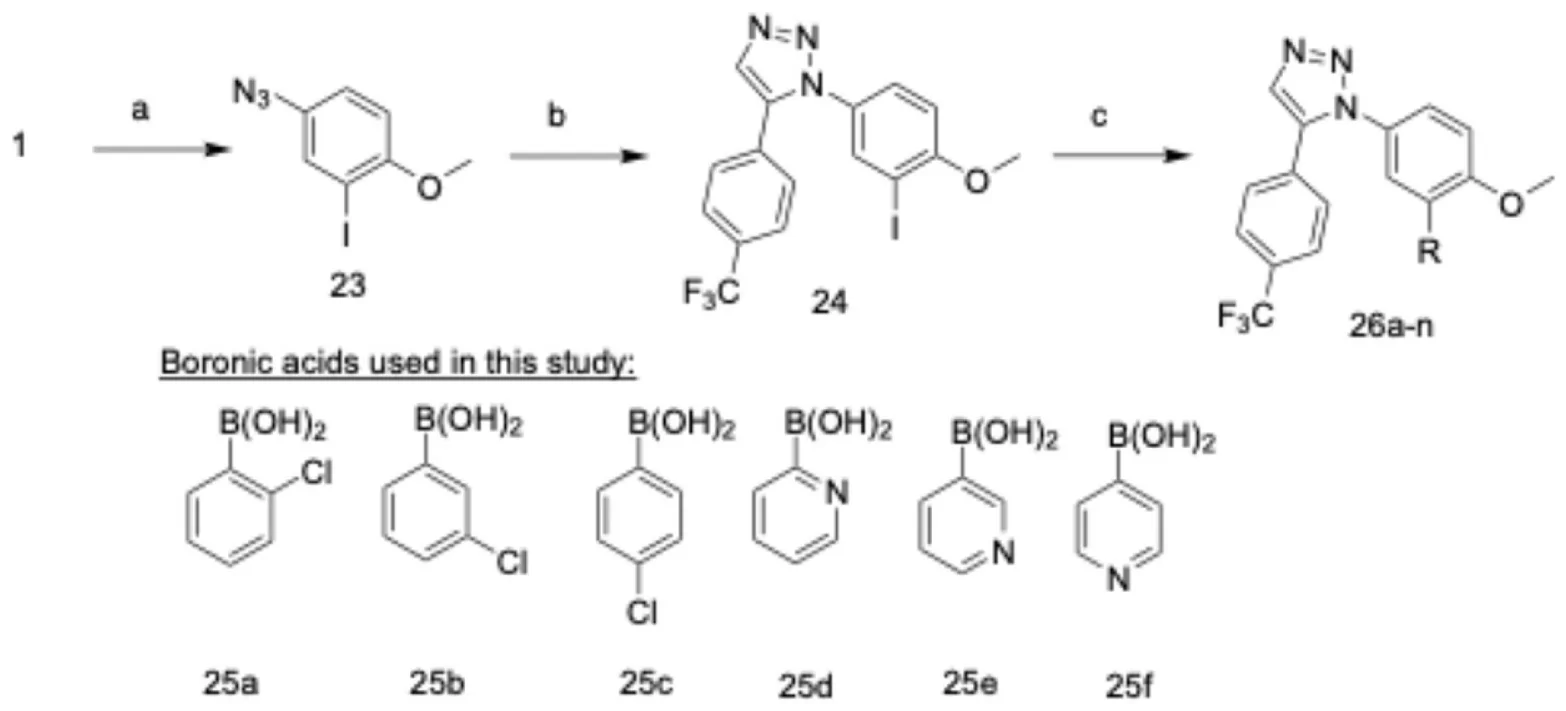
**1** was added to dilute H_2_SO_4_ at 0 °*C*, then aqueous NaNO_2_ was added to stir for 1 h. Then aqueous NaN_3_ was added to stir for 1 h. The reaction was allowed to stir for 12 h at 25 °*C*, 85%. B) **23** and **17** were dissolved in 1,4-dioxane, argon atmosphere, C_p_*RuCl(PPh_3_)_2_ (cat.), 80 °*C*, 16 h, 22%. c) **24**, 25a-n, K_2_CO_3_, Pd(dppf)Cl_2_, argon atmosphere, 1,4-dioxane, 120 °*C*,16 h 10-40%. *Pd(PPh_3_)_2_Cl_2_.

**Scheme 6.**
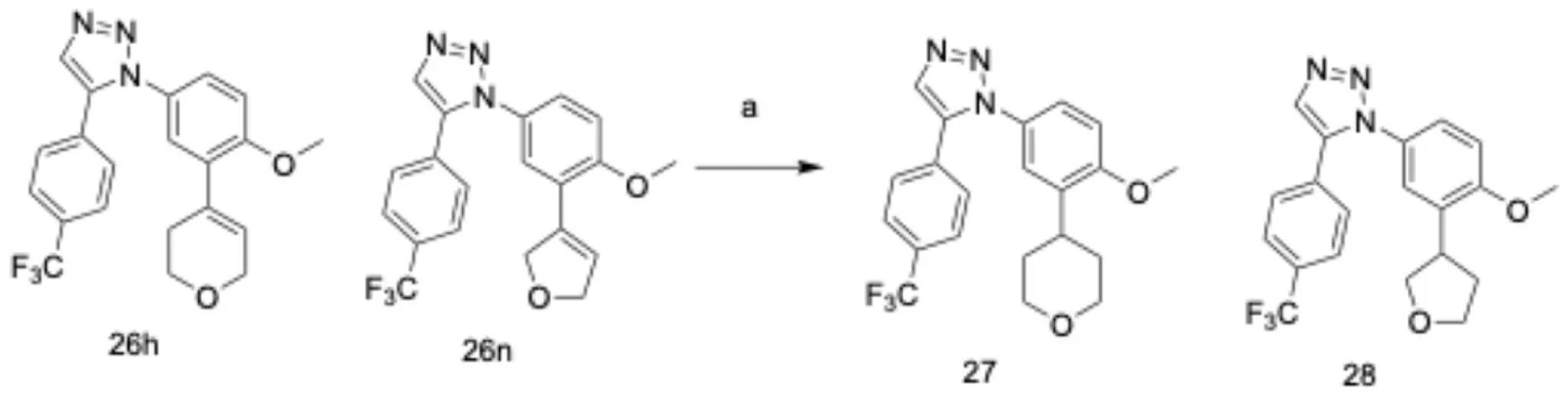
a) **26h** or **26o**, H_2_, Pd/C, EtOAc, 25 °*C*, 16 h, 40-50%.

**Scheme 7.**
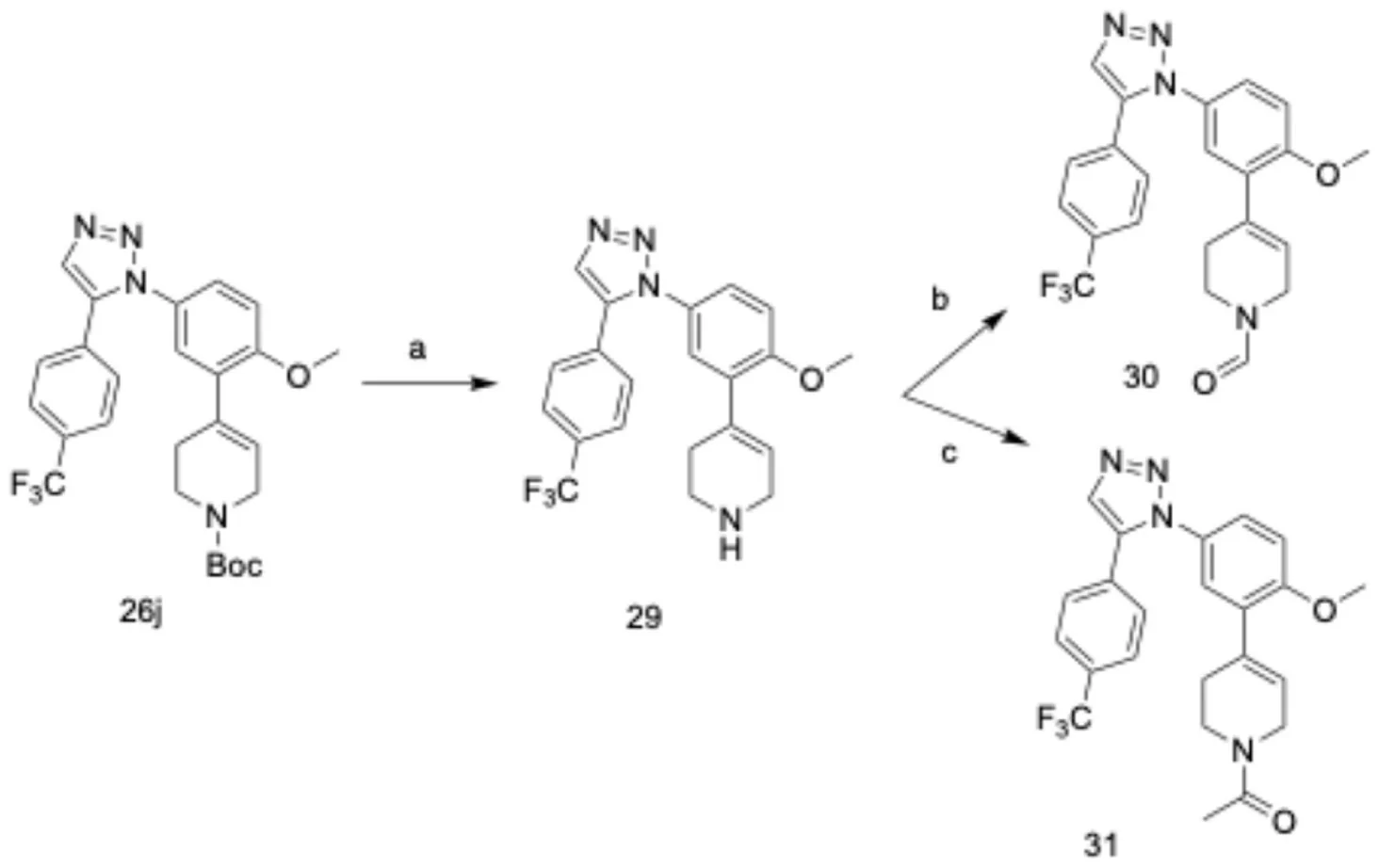
a) **26j**, 4 M HCl in dioxane, DCM, 25 °*C*, 16 h, 70%. b) **29**, ZnCl_2_, formic acid, 70 °*C*, 24 h, 35%. c) **29**, TEA, acetic anhydride 25 °*C*, 16 h, 31%.

**Scheme 8.**
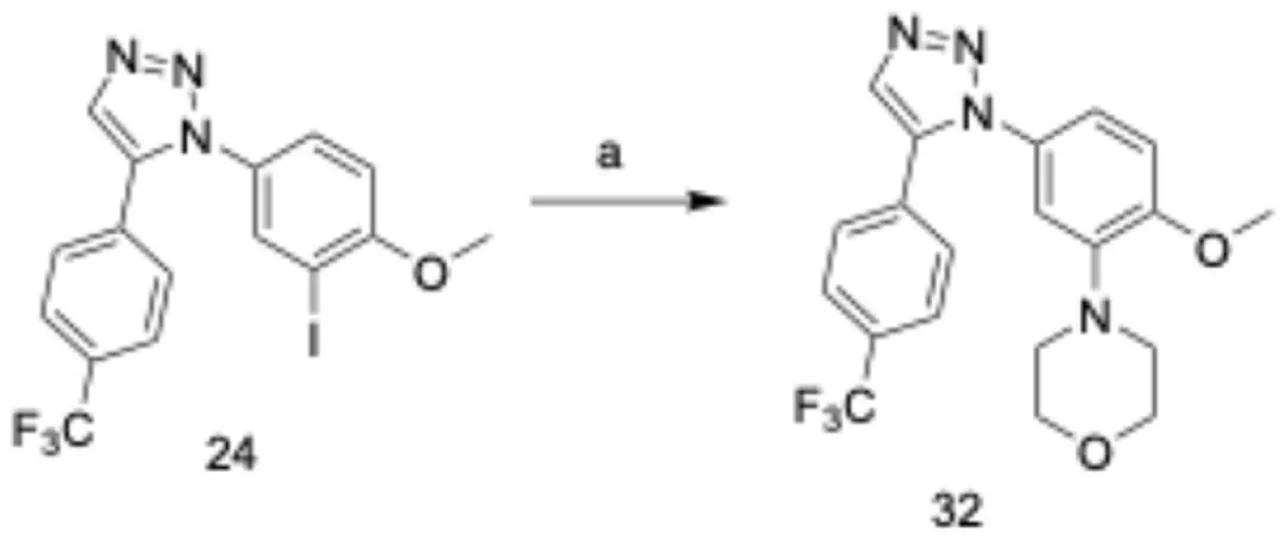
**24**, dimethylglycine, CuI, morpholine, argon atmosphere, DMF, 120 °*C*,16 h 25%.

**Scheme 9.**
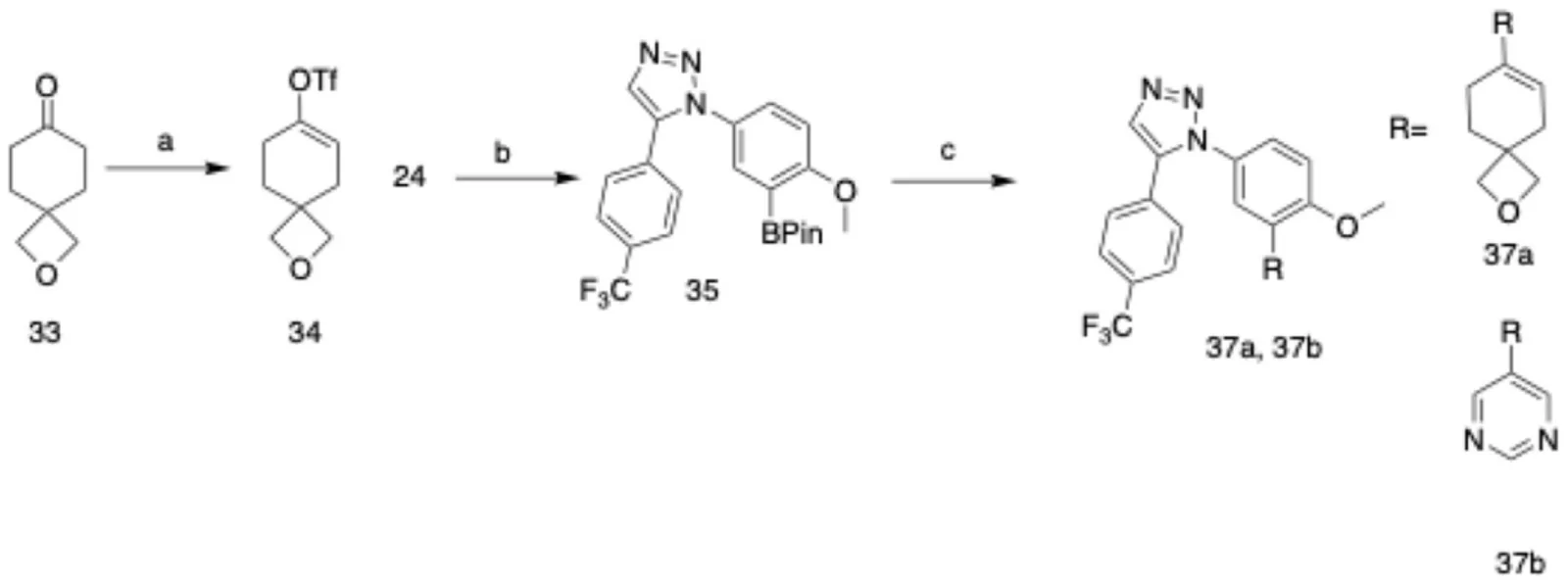
A) **33**, LDA, THF, −78 °*C*, 2 h 1,1,1-trifluoro-N-phenyl-N-((trifluoromethyl)sulfonyl)methanesulfonamide, 25 °*C*, 48 h, 33%. b) **24**, HBPin, SPhos, Pd (CH_3_CN)_2_Cl_2_, DIPEA, inert atmosphere, 80 °*C*, 24 h, 11.2% [13]. c) **35, 34** or **36**, K_2_CO_3_, Pd(dppf)Cl_2_, argon atmosphere, 1,4-dioxane, 120 °*C*,16 h 11-35%.

**Scheme 10.**
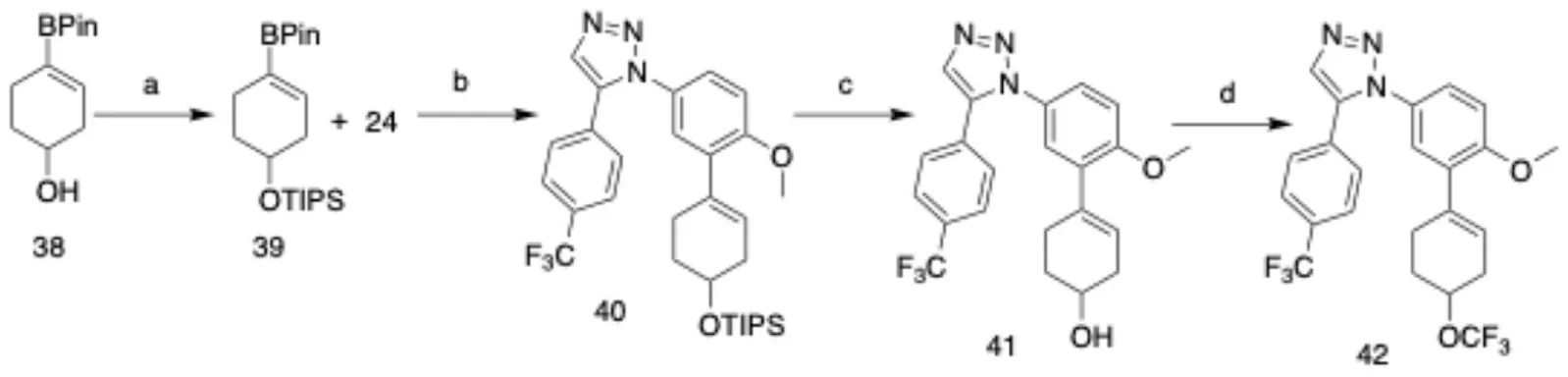
a) **38**, DIPEA, TIPS-OTf, DCM, 16 h, 25 °*C*, 71%. b) **24, 39**, K_2_CO_3_, Pd(PPh_3_)_2_Cl_2_, argon atmosphere, 1,4-dioxane, 120 °*C*,16 h 31%. c) **40**, TBAF, THF, 25 °*C*, 0.5 h, 50%. d) **41**, Togni reagent II, Zn(NOTf_2_)_2_, argon atmosphere CHCl_3_, 25 °*C*, 48 h, 74%.

**Scheme 11.**
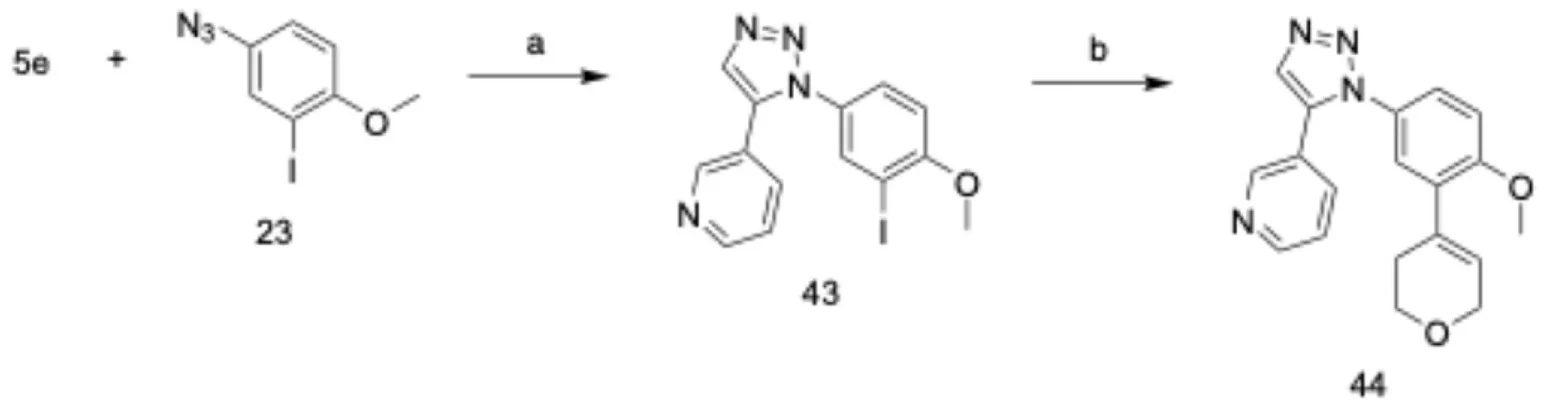
a) **5e** and **23** were dissolved in 1,4-dioxane, argon atmosphere, C_p_*RuCl(COD) (cat.), 80 °*C*, 16 h, 25%. B) **43**, 25h, K_2_CO_3_, Pd(PPh_3_)_2_Cl_2_, argon atmosphere, 1,4-dioxane, 120 °*C*,16 h 31%.

**Table 1:** IC_50_ data for phenyl ring A nitrogen scan and chlorinated derivatives by the split *renilla* luciferase assay.

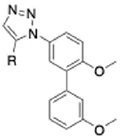		
Compound	Structure	IC_50_ μM
6a	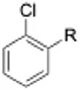	0.63 ± 0.04
6b	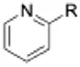	0.86 ± 0.3
6c	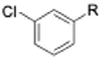	>5
6d	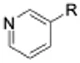	>5
6e	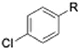	0.15 ± 0.02
6f	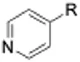	4.73 ± 0.3

**Table 2:** IC_50_ data for phenyl ring B nitrogen scan and chlorinated derivatives by the split *renilla* luciferase assay.

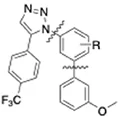		
Compound	Structure	IC_50_ μM
13*	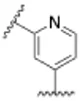	~5
18a	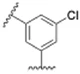	>5
18b	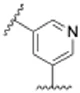	>5
18c	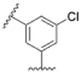	>5
18d	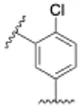	>5
22	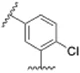	1.4 ± 1

**Table 3:** IC_50_ data for phenyl ring C nitrogen scan and chlorinated derivatives by the split *renilla* luciferase assay.

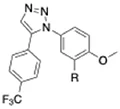		
Compound	Structure	IC_50_ μM
26a	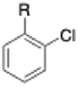	1.7 ± 0.7
26b	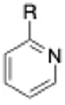	0.87 ± 0.3
26c	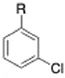	>5
26d	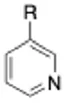	0.53 ± 0.02
26e	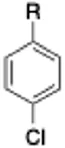	0.27 ± 0.05
26f	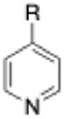	0.15 ± 0.02

**Table 4:** IC_50_ data for C ring derivatives by the split *renilla* luciferase assay.

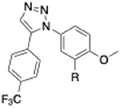		
Compound	Structure	IC_50_ μM
26g	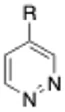	>5
26h	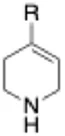	0.050 ± 0.001
26i	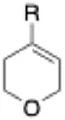	0.29 ± 0.04
26k	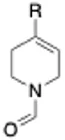	0.46 ± 0.2
26l	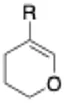	0.041 ± 0.007
26 m	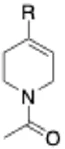	0.49 ± 0.005
26n	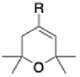	>5
27	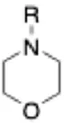	>5
28	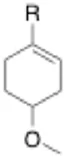	>5
29	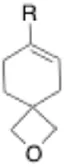	>5
30	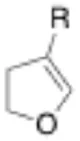	>5
31	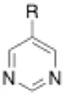	>5
32	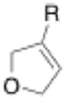	>5
37a	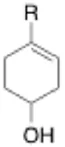	0.26 ± 0.02
37b	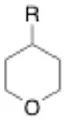	0.052 ± 0.01
41	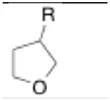	>5
42	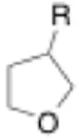	>5

**Table 5:** Lead triazole A and two new efficacious molecules **26l** and **37b** were evaluated for physiochemical properties solubility in 5% DMSO, half life with human liver microsomes (*h*LM), and apparent permeability and efflux ratio (ER) with MDCK-MDR1 monolayers. Molecular weight (MW), topological polar surface area (tPSA) and the cLogP were calculated/predicted by ChemDraw.

Compound	MW	tPSA	cLogP	Solubility (μM)	*h*LM t_1/2_ (min)	P_app A- > B_ (nm/s)	ER
Triazole A	425.41	46.42	5.836	5.0 ± 0.1	249.3 ± 1	[1.8 ± 0.001]x10^−6^	0.61 ± 0.01
**26l**	429.44	46.42	5.534	17.0 ± 0.3	22.8 ± 9	[7.5 ± 1]x10^−6^	0.35 ± 0.1
**37b**	397.36	61.91	2.923	203.1 ± 17	844.7 ± 42	[9.5 ± 1]x10^−6^	0.07 ± 0.01

## Data Availability

Data will be made available on request.

## Appendix B. Supplementary data

Supplementary data to this article can be found online at https://doi.org/10.1016/j.ejmech.2026.118612.

## Abbreviations

Hsp90: the 90 kDa heat shock protein
Aha1: Activator of Hsp90 ATPase activity homolog 1
PgP: P-glycoprotein
co-IP: co-immunoprecipitation
ER: efflux ratio
MW: molecular weight
tPSA: topological polar surface area
*h*LM t_1/2_: human liver microsomal half life
