# Supplementary material for: Optimization of triazole-based small molecule disruptors of the Aha1/Hsp90 complex that manifest potent activities
Source: Eur J Med Chem. Author manuscript; Available in PMC 2026 Nov 2. (PMC13726759; doi:10.1016/j.ejmech.2026.118612)
Supplement: Supplementary file 1 [file NIHMS2216523-supplement-Supplemental_Data.docx]

**SUPPORTING INFORMATION**

***Optimization of triazole-based small molecule disruptors of the Aha1/Hsp90 complex that manifest potent activities***

Kevin C. Catalfano, Margaret H. Rakonick and Brian S. J. Blagg^*^

Department of Chemistry and Biochemistry, Warren Family Research Center for Drug Discovery, University of Notre Dame. Notre Dame, Indiana, 46556, USA.

^*^corresponding author: [bblagg@nd.edu](mailto:bblagg@nd.edu)

Table of Contents

^1^H, ^13^C NMR and HRMS data for all compounds……………………………...……………...3-24

NMR data **26f** and **26h**……………………………………………………………………….25-26

HPLC trace **26f** and **26h**……………………………………………….………………………...27

SK-Br-3 co-IP Western blots……………………………………………………………………..28

Lucifer Yellow measurements……………..……………………………………………….…….29

*3',6-dimethoxy-[1,1'-biphenyl]-3-amine* (**3**)

^1^H NMR (400 MHz, Chloroform-*d*) δ 7.32 (t, *J* = 7.9 Hz, 1H), 7.14 – 7.04 (m, 2H), 6.88 (ddd, *J* = 8.2, 2.6, 1.0 Hz, 1H), 6.83 (d, *J* = 8.6 Hz, 1H), 6.72 (d, *J* = 2.8 Hz, 1H), 6.67 (dd, *J* = 8.6, 2.9 Hz, 1H), 3.84 (s, 3H), 3.72 (s, 3H), 3.49 (s, 2H).

*5-azido-2,3'-dimethoxy-1,1'-biphenyl* (**4**)

^1^H NMR (400 MHz, Chloroform-*d*) δ 7.35 (t, *J* = 7.9 Hz, 1H), 7.13 – 7.05 (m, 2H), 7.03 – 6.96 (m, 3H), 6.96 – 6.89 (m, 1H), 3.85 (s, 3H), 3.80 (s, 3H).

**General procedure for synthesis of compounds 6a-f**

*5-(2-chlorophenyl)-1-(3',6-dimethoxy-[1,1'-biphenyl]-3-yl)-1H-1,2,3-triazole* (**6a**)

^1^H NMR (400 MHz, Chloroform-*d*) δ 7.94 – 7.82 (m, 1H), 7.54 – 7.36 (m, 3H), 7.36 – 7.29 (m, 2H), 7.29 – 7.26 (m, 2H), 7.24 (dd, *J* = 4.5, 2.6 Hz, 1H), 7.03 – 6.75 (m, 4H), 3.83 (d, *J* = 3.3 Hz, 3H), 3.81 – 3.75 (m, 2H). ^13^C NMR (101 MHz, CDCl_3_) δ 159.24, 156.71, 138.22, 134.71, 134.29, 132.10, 131.09, 130.99, 130.22, 129.61, 129.05, 127.02, 126.84, 126.81, 126.66, 124.46, 121.87, 115.04, 113.15, 111.45, 55.85, 55.32. HRMS-ESI: calcd. for C_22_H_19_ClN_3_O_2_, 392.1160 [M+1]+; found 392.1150. Purity 95.4%

*5-(3-chlorophenyl)-1-(3',6-dimethoxy-[1,1'-biphenyl]-3-yl)-1H-1,2,3-triazole* (**6b**)

^1^H NMR (400 MHz, Chloroform-*d*) δ 7.82 (s, 1H), 7.32 – 7.24 (m, 3H), 7.22 (dd, *J* = 8.7, 3.2 Hz, 2H), 7.06 (dt, *J* = 7.7, 1.4 Hz, 1H), 6.99 – 6.90 (m, 2H), 6.86 (t, *J* = 2.0 Hz, 1H), 6.81 (ddd, *J* = 8.2, 2.7, 1.0 Hz, 1H), 3.80 (s, 3H), 3.73 (s, 3H), 1.64 – 1.40 (m, 5H), 1.22 – 1.12 (m, 6H), 0.91 – 0.58 (m, 4H). ^13^C NMR (101 MHz, CDCl_3_) δ 159.42, 157.22, 135.01, 133.62, 131.68, 130.29, 129.45, 129.34, 129.26, 128.84, 128.73, 127.68, 126.89, 125.46, 122.01, 115.08, 113.49, 111.80, 56.09, 55.42. HRMS-ESI: calcd. for C_22_H_19_ClN_3_O_2_, 392.1160 [M+1]+; found 392.1150. Purity 90.5%

*5-(4-chlorophenyl)-1-(3',6-dimethoxy-[1,1'-biphenyl]-3-yl)-1H-1,2,3-triazole* (**6c**)

1H NMR (400 MHz, Chloroform-*d*) δ 7.86 (s, 1H), 7.40 – 7.34 (m, 2H), 7.34 – 7.27 (m, 3H), 7.25 – 7.18 (m, 2H), 7.05 – 6.96 (m, 2H), 6.94 – 6.84 (m, 2H), 3.87 (s, 3H), 3.80 (s, 3H). ^13^C NMR (101 MHz, CDCl_3_) δ 159.40, 157.13, 138.13, 136.71, 135.53, 133.37, 131.60, 129.99, 129.38, 129.32, 129.23, 127.67, 125.51, 125.42, 121.98, 114.94, 113.53, 111.75, 56.03, 55.38. HRMS-ESI: calcd. for C_22_H_19_ClN_3_O_2_, 392.1160 [M+1]+; found 392.1160. Purity 92.7%

*2-(1-(3',6-dimethoxy-[1,1'-biphenyl]-3-yl)-1H-1,2,3-triazol-5-yl)pyridine* (**6d**)

^1^H NMR (400 MHz, Chloroform-*d*) δ 8.74 – 8.55 (m, 1H), 8.12 (d, *J* = 2.9 Hz, 1H), 7.68 (tt, *J* = 7.8, 2.4 Hz, 1H), 7.41 (dt, *J* = 8.6, 2.8 Hz, 1H), 7.34 (d, *J* = 2.9 Hz, 1H), 7.29 (ddd, *J* = 8.3, 5.8, 2.9 Hz, 2H), 7.11 – 6.93 (m, 3H), 6.93 – 6.79 (m, 1H), 3.87 (d, *J* = 3.1 Hz, 3H), 3.80 (d, *J* = 2.8 Hz, 2H). ^13^C NMR (101 MHz, CDCl_3_) δ 159.37, 157.16, 150.27, 146.93, 138.35, 137.31, 136.80, 134.54, 131.35, 130.04, 129.21, 127.97, 125.66, 123.67, 123.47, 122.01, 115.27, 113.23, 111.60, 56.06, 55.42. HRMS-ESI: calcd. for C_21_H_19_N_4_O_2_, 359.1503 [M+1]+; found 359.1505. Purity 94.8%

*3-(1-(3',6-dimethoxy-[1,1'-biphenyl]-3-yl)-1H-1,2,3-triazol-5-yl)pyridine* (**6e**)

^1^H NMR (400 MHz, Chloroform-*d*) δ 9.08 (dd, *J* = 2.2, 0.9 Hz, 1H), 8.61 (dd, *J* = 4.9, 1.7 Hz, 1H), 8.29 (dt, *J* = 7.9, 1.9 Hz, 1H), 8.23 (s, 1H), 7.75 (dd, *J* = 8.7, 2.7 Hz, 1H), 7.71 (d, *J* = 2.8 Hz, 1H), 7.43 – 7.40 (m, 1H), 7.40 – 7.35 (m, 1H), 7.17 – 7.11 (m, 3H), 6.94 (ddd, *J* = 8.3, 2.6, 1.0 Hz, 1H), 3.90 (s, 3H), 3.86 (s, 3H). ^13^C NMR (101 MHz, CDCl_3_) δ 159.53, 157.05, 149.62, 147.27, 138.40, 133.28, 132.07, 130.43, 129.42, 123.99, 123.51, 122.05, 121.26, 118.34, 115.48, 115.16, 113.32, 112.12, 99.28, 56.21, 55.50. HRMS-ESI: calcd. for C_21_H_19_N_4_O_2_, 359.1503 [M+1]+; found 359.1505. Purity 95.4%

*4-(1-(3',6-dimethoxy-[1,1'-biphenyl]-3-yl)-1H-1,2,3-triazol-5-yl)pyridine* (**6f**)

^1^H NMR (400 MHz, Chloroform-*d*) δ 8.73 – 8.66 (m, 2H), 8.28 (s, 1H), 7.82 – 7.77 (m, 2H), 7.75 (dd, *J* = 8.8, 2.7 Hz, 1H), 7.70 (d, *J* = 2.7 Hz, 1H), 7.40 – 7.34 (m, 1H), 7.17 – 7.09 (m, 3H), 6.94 (ddd, *J* = 8.3, 2.6, 1.0 Hz, 1H), 3.90 (s, 3H), 3.86 (s, 3H). ^13^C NMR (101 MHz, CDCl_3_) δ 159.53, 157.14, 150.69, 145.91, 138.34, 137.88, 132.09, 130.30, 129.43, 123.50, 122.02, 121.28, 120.15, 119.48, 115.50, 113.30, 112.14, 56.21, 55.50. HRMS-ESI: calcd. for C_21_H_19_N_4_O_2_, 359.1503 [M+1]+; found 359.1504. Purity 92.8%

**General procedure for the synthesis of 13**

*methyl 4-(3-methoxyphenyl)picolinate* (**8**)

^1^H NMR (400 MHz, Chloroform-*d*) δ 8.78 (dd, *J* = 5.1, 0.8 Hz, 1H), 8.37 (dd, *J* = 1.9, 0.8 Hz, 1H), 7.68 (dd, *J* = 5.1, 1.9 Hz, 1H), 7.43 (t, *J* = 8.0 Hz, 1H), 7.28 (dd, *J* = 1.7, 0.9 Hz, 1H), 7.20 (t, *J* = 2.1 Hz, 1H), 7.02 (ddd, *J* = 8.3, 2.6, 0.9 Hz, 1H), 4.05 (s, 3H), 3.89 (s, 3H).

*4-(3-methoxyphenyl)picolinic acid* (**9**)

^1^H NMR (400 MHz, Chloroform-*d*) δ 8.66 (d, *J* = 5.2 Hz, 1H), 8.49 – 8.44 (m, 1H), 7.79 (dd, *J* = 5.1, 1.8 Hz, 1H), 7.44 (t, *J* = 8.0 Hz, 1H), 7.30 – 7.27 (m, 1H), 7.21 (t, *J* = 2.1 Hz, 1H), 7.04 (dt, *J* = 8.3, 1.7 Hz, 1H), 3.89 (s, 3H).

*4-(3-methoxyphenyl)-N-(4-(trifluoromethyl)phenyl)picolinamide* (**12**)

^1^H NMR (400 MHz, Chloroform-*d*) δ 8.83 (dd, *J* = 5.1, 0.8 Hz, 1H), 8.47 (dd, *J* = 1.8, 0.8 Hz, 1H), 7.74 – 7.63 (m, 3H), 7.44 (t, *J* = 8.0 Hz, 1H), 7.30 (ddd, *J* = 7.7, 1.8, 0.9 Hz, 1H), 7.22 (dd, *J* = 2.5, 1.7 Hz, 1H), 7.18 – 7.10 (m, 2H), 7.02 (ddd, *J* = 8.3, 2.5, 0.9 Hz, 1H), 3.89 (s, 3H).

*4-(3-methoxyphenyl)-2-(1-(4-(trifluoromethyl)phenyl)-1H-tetrazol-5-yl)pyridine* (**13**)

^1^H NMR (400 MHz, Chloroform-*d*) δ 8.54 – 8.42 (m, 2H), 7.80 (d, *J* = 8.4 Hz, 2H), 7.70 – 7.63 (m, 2H), 7.63 – 7.59 (m, 1H), 7.45 (t, *J* = 8.0 Hz, 1H), 7.29 (ddd, *J* = 7.7, 1.7, 0.9 Hz, 1H), 7.22 (t, *J* = 2.1 Hz, 1H), 7.04 (ddd, *J* = 8.3, 2.6, 0.9 Hz, 1H), 3.90 (s, 3H). ^13^C NMR (101 MHz, CDCl_3_) δ 160.49, 152.86, 150.19, 150.15, 144.45, 138.38, 138.31, 130.62, 126.50, 126.46, 126.42, 126.40, 123.77, 123.14, 119.59, 115.44, 112.84, 55.63. HRMS-ESI: calcd. for C_20_H_15_F_3_N_5_O, 398.1223 [M+1]+; found 398.1221. Purity 91.0%

**General procedure for the synthesis of 18a**

*5-(3-methoxyphenyl)pyridin-3-amine* (**15a**)

^1^H NMR (400 MHz, Chloroform-*d*) δ 8.24 (s, 1H), 8.08 (s, 1H), 7.36 (t, *J* = 7.9 Hz, 1H), 7.18 – 7.00 (m, 3H), 6.92 (dd, *J* = 8.2, 2.4 Hz, 1H), 4.24 – 3.89 (m, 1H), 3.85 (s, 3H).

*3-azido-5-(3-methoxyphenyl)pyridine* (**16a**)

^1^H NMR (400 MHz, Chloroform-*d*) δ 8.51 (d, *J* = 1.9 Hz, 1H), 8.24 (d, *J* = 2.5 Hz, 1H), 7.38 (t, *J* = 2.3 Hz, 1H), 7.30 (t, *J* = 7.9 Hz, 1H), 7.04 (dt, *J* = 7.6, 1.2 Hz, 1H), 6.98 (t, *J* = 2.1 Hz, 1H), 6.87 (dd, *J* = 8.2, 2.6 Hz, 1H), 3.77 (s, 3H).

*3-(3-methoxyphenyl)-5-(5-(4-(trifluoromethyl)phenyl)-1H-1,2,3-triazol-1-yl)pyridine* (**18a**)

^1^H NMR (400 MHz, Chloroform-*d*) δ 8.92 (dd, *J* = 26.0, 2.2 Hz, 2H), 8.32 (s, 2H), 8.00 (d, *J* = 8.1 Hz, 2H), 7.68 (d, *J* = 8.2 Hz, 2H), 7.39 (t, *J* = 8.0 Hz, 1H), 7.12 (t, *J* = 2.2 Hz, 1H), 6.96 (dd, *J* = 8.3, 2.5 Hz, 1H), 3.90 – 3.81 (m, 3H). ^13^C NMR (101 MHz, CDCl_3_) δ 193.91, 160.49, 148.84, 147.77, 140.04, 137.99, 137.60, 133.71, 133.35, 130.66, 126.57, 126.27, 126.22, 126.18, 119.85, 118.46, 114.49, 113.29, 55.62. HRMS-ESI: calcd. for C_21_H_16_F_3_N_4_O, 397.1271 [M+1]+; found 397.1271. Purity 95.2%

**General procedure for the synthesis of compounds 18b-d**

*4-chloro-3'-methoxy-[1,1'-biphenyl]-3-amine* (**15b**)

^1^H NMR (400 MHz, Chloroform-*d*) δ 7.25 (t, *J* = 7.9 Hz, 1H), 7.20 (s, 1H), 7.04 (ddd, *J* = 7.6, 1.7, 0.9 Hz, 1H), 6.98 (dd, *J* = 2.6, 1.7 Hz, 1H), 6.89 (d, *J* = 2.1 Hz, 1H), 6.87 – 6.77 (m, 2H), 4.03 (s, 2H), 3.78 (s, 3H).

*5-chloro-3'-methoxy-[1,1'-biphenyl]-3-amine* (**15c**)

^1^H NMR (400 MHz, Chloroform-*d*) δ 7.33 (t, *J* = 7.9 Hz, 1H), 7.11 (ddd, *J* = 7.7, 1.8, 1.0 Hz, 1H), 7.05 (dd, *J* = 2.6, 1.7 Hz, 1H), 6.95 (t, *J* = 1.7 Hz, 1H), 6.90 (ddd, *J* = 8.2, 2.6, 0.9 Hz, 1H), 6.80 – 6.56 (m, 2H), 3.86 (s, 3H), 3.84 – 3.74 (m, 2H).

*6-chloro-3'-methoxy-[1,1'-biphenyl]-3-amine* (**15d**)

^1^H NMR (400 MHz, Chloroform-*d*) δ 7.22 (t, *J* = 7.9 Hz, 1H), 7.11 (d, *J* = 8.5 Hz, 1H), 6.94 – 6.84 (m, 2H), 6.81 (ddd, *J* = 8.3, 2.6, 1.0 Hz, 1H), 6.54 (d, *J* = 2.8 Hz, 1H), 6.48 (dd, *J* = 8.5, 2.8 Hz, 1H), 3.72 (s, 3H), 3.62 (s, 1H).

*3-azido-4-chloro-3'-methoxy-1,1'-biphenyl* (**16b**)

^1^H NMR (400 MHz, Chloroform-*d*) δ 7.24 (td, *J* = 13.0, 11.6, 4.7 Hz, 2H), 7.16 (t, *J* = 2.1 Hz, 1H), 7.11 (dt, *J* = 8.3, 2.2 Hz, 1H), 7.04 – 6.87 (m, 2H), 6.79 (dd, *J* = 8.1, 2.8 Hz, 1H), 3.87 – 3.56 (m, 3H).

*3-azido-5-chloro-3'-methoxy-1,1'-biphenyl* (**16c**)

^1^H NMR (400 MHz, Chloroform-*d*) δ 7.42 – 7.29 (m, 2H), 7.15 – 7.07 (m, 2H), 7.03 (dt, *J* = 22.9, 2.1 Hz, 2H), 6.95 (ddd, *J* = 8.2, 2.5, 0.9 Hz, 1H), 3.87 (s, 3H).

*5-azido-2-chloro-3'-methoxy-1,1'-biphenyl* (**16d**)

^1^H NMR (400 MHz, Chloroform-*d*) δ 7.34 (d, *J* = 8.5 Hz, 1H), 7.27 (td, *J* = 7.6, 1.2 Hz, 1H), 6.94 – 6.88 (m, 2H), 6.88 – 6.85 (m, 2H), 6.85 – 6.82 (m, 1H), 3.75 (s, 3H).

*1-(4-chloro-3'-methoxy-[1,1'-biphenyl]-3-yl)-5-(4-(trifluoromethyl)phenyl)-1H-1,2,3-triazole* (**18b**)

^1^H NMR (400 MHz, Chloroform-*d*) δ 8.01 (s, 1H), 7.83 – 7.47 (m, 5H), 7.38 (d, *J* = 8.2 Hz, 3H), 7.21 – 7.02 (m, 2H), 6.95 (d, *J* = 8.3 Hz, 1H), 3.85 (s, 3H), 0.92 (d, *J* = 30.8 Hz, 1H). ^13^C NMR (101 MHz, CDCl_3_) δ 160.22, 141.45, 139.43, 138.08, 134.48, 132.95, 130.31, 130.27, 130.21, 128.01, 127.68, 126.06, 126.03, 125.99, 125.95, 122.28, 119.41, 113.95, 112.78, 55.42. HRMS-ESI: calcd. for C_22_H_16_F_3_N_3_O, 430.0929 [M+1]+; found 430.0946. Purity 94.0%

*1-(5-chloro-3'-methoxy-[1,1'-biphenyl]-3-yl)-5-(4-(trifluoromethyl)phenyl)-1H-1,2,3-triazole* (**18c**)

^1^H NMR (400 MHz, Chloroform-*d*) δ 7.88 (s, 1H), 7.65 – 7.57 (m, 2H), 7.39 – 7.34 (m, 2H), 7.30 – 7.27 (m, 1H), 7.27 – 7.22 (m, 2H), 7.16 (d, *J* = 3.1 Hz, 1H), 7.09 (dd, *J* = 8.9, 3.3 Hz, 2H), 6.87 (ddd, *J* = 8.5, 5.1, 1.9 Hz, 2H), 6.80 (t, *J* = 2.1 Hz, 1H), 3.74 (s, 3H).^13^C NMR (101 MHz, CDCl_3_) δ 160.14, 148.96, 148.32, 144.14, 135.69, 134.10, 130.22, 129.05, 128.39, 127.96, 126.17, 126.14, 126.10, 123.79, 122.03, 120.36, 119.41, 114.33, 112.57, 55.35. HRMS-ESI: calcd. for C_22_H_16_ClF_3_N_3_O, 430.0929 [M+1]+; found 430.0926. Purity 90.7%

*1-(6-chloro-3'-methoxy-[1,1'-biphenyl]-3-yl)-5-(4-(trifluoromethyl)phenyl)-1H-1,2,3-triazole* (**18d**)

1H NMR (400 MHz, Chloroform-*d*) δ 7.93 (s, 1H), 7.69 (d, *J* = 8.2 Hz, 2H), 7.57 (d, *J* = 8.5 Hz, 1H), 7.44 – 7.40 (m, 2H), 7.34 (d, *J* = 2.5 Hz, 1H), 7.33 – 7.30 (m, 1H), 7.30 – 7.28 (m, 1H), 6.93 (ddd, *J* = 8.3, 2.6, 1.0 Hz, 1H), 6.87 (dt, *J* = 7.5, 1.2 Hz, 1H), 6.84 (dd, *J* = 2.6, 1.6 Hz, 1H), 3.80 (s, 3H). ^13^C NMR (101 MHz, CDCl_3_) δ 159.35, 141.85, 138.87, 136.36, 134.69, 134.07, 133.83, 131.23, 129.37, 129.07, 127.63, 126.16, 126.13, 126.09, 126.05, 124.72, 121.57, 114.73, 114.09, 55.31. HRMS-ESI: calcd. for C_22_H_16_ClF_3_N_3_O, 430.092851 [M+1]+; found 430.094607. Purity 93.4%

**General procedure for the synthesis of 22**

*4-azido-2-bromopyridine* (**20**)

^1^H NMR (400 MHz, Chloroform-*d*) δ 8.28 (d, *J* = 5.5 Hz, 1H), 7.15 (d, *J* = 2.0 Hz, 1H), 6.90 (dd, *J* = 5.5, 2.0 Hz, 1H).

*2-bromo-4-(5-(4-(trifluoromethyl)phenyl)-1H-1,2,3-triazol-1-yl)pyridine* (**21**)

^1^H NMR (400 MHz, Chloroform-*d*) δ 8.57 (t, *J* = 4.6 Hz, 1H), 8.44 (dd, *J* = 5.5, 2.6 Hz, 1H), 8.39 (d, *J* = 5.8 Hz, 1H), 7.80 (dd, *J* = 5.5, 1.9 Hz, 1H), 7.74 (dd, *J* = 8.2, 1.7 Hz, 2H), 7.42 (d, *J* = 8.0 Hz, 2H).

*2-(3-methoxyphenyl)-4-(5-(4-(trifluoromethyl)phenyl)-1H-1,2,3-triazol-1-yl)pyridine* (**22**)

^1^H NMR (400 MHz, Chloroform-*d*) δ 8.87 (d, *J* = 5.4 Hz, 1H), 8.44 (s, 1H), 8.22 (d, *J* = 2.0 Hz, 1H), 8.07 (d, *J* = 8.1 Hz, 2H), 7.75 (d, *J* = 8.1 Hz, 2H), 7.72 – 7.59 (m, 3H), 7.45 (t, *J* = 7.9 Hz, 1H), 7.11 – 6.99 (m, 1H), 3.93 (s, 3H). ^13^C NMR (101 MHz, CDCl_3_) δ 160.39, 159.94, 151.66, 147.84, 143.89, 139.67, 130.17, 126.31, 126.25, 126.24, 126.20, 126.16, 119.57, 117.76, 116.15, 112.44, 112.29, 110.81, 55.63. HRMS-ESI: calcd. for C_21_H_19_N_4_O_2_, 397.1271 [M+1]+; found 397.1260. Purity 97.4%

*4-azido-2-iodo-1-methoxybenzene* (**23**)

^1^H NMR (400 MHz, Chloroform-*d*) δ 7.45 (d, *J* = 2.7 Hz, 1H), 6.98 (dd, *J* = 8.7, 2.7 Hz, 1H), 6.79 (d, *J* = 8.8 Hz, 1H), 3.87 (s, 3H).

*1-(3-iodo-4-methoxyphenyl)-5-(4-(trifluoromethyl)phenyl)-1H-1,2,3-triazole* (**24**)

^1^H NMR (400 MHz, Chloroform-*d*) δ 7.94 – 7.85 (m, 2H), 7.64 (d, *J* = 8.1 Hz, 2H), 7.40 – 7.33 (m, 2H), 7.21 (dd, *J* = 8.8, 2.6 Hz, 1H), 6.83 (d, *J* = 8.8 Hz, 1H), 3.94 (s, 3H).

*1-(2'-chloro-6-methoxy-[1,1'-biphenyl]-3-yl)-5-(4-(trifluoromethyl)phenyl)-1H-1,2,3-triazole* (**26a**)

^1^H NMR (400 MHz, Chloroform-*d*) δ 7.93 (s, 1H), 7.63 (d, *J* = 8.1 Hz, 2H), 7.45 – 7.39 (m, 4H), 7.31 – 7.26 (m, 2H), 7.18 – 7.14 (m, 1H), 7.10 (d, *J* = 2.7 Hz, 1H), 7.06 (d, *J* = 8.8 Hz, 1H), 3.84 (s, 3H). ^13^C NMR (101 MHz, CDCl_3_) δ 157.74, 136.65, 135.88, 133.74, 133.66, 131.52, 129.69, 129.60, 129.34, 129.05, 128.83, 128.36, 126.68, 126.47, 126.09, 126.05, 126.02, 125.98, 111.74, 56.15. HRMS-ESI: calcd. for C_22_H_16_ClF_3_N_3_O, 430.0929 [M+1]+; found 430.0930. Purity 96.7%

*1-(3'-chloro-6-methoxy-[1,1'-biphenyl]-3-yl)-5-(4-(trifluoromethyl)phenyl)-1H-1,2,3-triazole* (**26b**)

^1^H NMR (400 MHz, Chloroform-*d*) δ 7.93 (s, 1H), 7.67 (d, *J* = 8.1 Hz, 2H), 7.45 – 7.39 (m, 3H), 7.33 – 7.30 (m, 4H), 7.29 (d, *J* = 2.7 Hz, 1H), 7.04 (d, *J* = 8.8 Hz, 1H), 3.88 (s, 3H).^13^C NMR (101 MHz, CDCl_3_) δ 157.07, 138.36, 136.35, 134.02, 133.72, 130.52, 130.32, 129.53, 129.42, 129.24, 128.95, 127.80, 127.52, 125.99, 125.95, 125.91, 125.79, 111.76, 77.35, 77.24, 77.04, 76.72, 56.00. HRMS-ESI: calcd. for C_22_H_16_ClF_3_N_3_O, 430.0929 [M+1]+; found 430.0940. Purity 95.7%

*1-(4'-chloro-6-methoxy-[1,1'-biphenyl]-3-yl)-5-(4-(trifluoromethyl)phenyl)-1H-1,2,3-triazole* (**26c**)

^1^H NMR (400 MHz, Chloroform-*d*) δ 7.93 (s, 1H), 7.66 (d, *J* = 8.1 Hz, 2H), 7.41 (d, *J* = 8.1 Hz, 2H), 7.38 – 7.28 (m, 5H), 7.25 (s, 1H), 7.02 (d, *J* = 8.8 Hz, 1H), 3.87 (s, 3H). ^13^C NMR (101 MHz, CDCl_3_) δ 157.19, 136.48, 135.15, 133.91, 133.80, 130.81, 130.67, 129.35, 129.10, 128.52, 127.51, 126.14, 126.10, 126.06, 126.03, 125.68, 111.87, 56.09. HRMS-ESI: calcd. for C_22_H_16_ClF_3_N_3_O, 430.0929 [M+1]+; found 430.0922. Purity 93.3%

*2-(2-methoxy-5-(5-(4-(trifluoromethyl)phenyl)-1H-1,2,3-triazol-1-yl)phenyl)pyridine* (**26d**)

^1^H NMR (400 MHz, Chloroform-*d*) δ 8.72 – 8.61 (m, 1H), 7.97 (d, *J* = 2.8 Hz, 1H), 7.92 (s, 1H), 7.84 (d, *J* = 8.0 Hz, 1H), 7.73 (td, *J* = 7.8, 1.9 Hz, 1H), 7.61 (d, *J* = 8.1 Hz, 2H), 7.43 (d, *J* = 8.1 Hz, 2H), 7.23 (dd, *J* = 8.7, 2.9 Hz, 2H), 7.03 (d, *J* = 8.8 Hz, 1H), 3.93 (s, 3H). ^13^C NMR (101 MHz, CDCl_3_) δ 207.01, 149.71, 149.69, 136.09, 134.13, 133.76, 129.03, 128.47, 126.52, 126.04, 126.00, 125.97, 125.14, 122.56, 114.89, 112.14, 112.02, 106.61, 56.16. HRMS-ESI: calcd. for C_21_H_16_F_3_N_4_O, 397.1271 [M+1]+; found 397.1264. Purity 90.9%

*3-(2-methoxy-5-(5-(4-(trifluoromethyl)phenyl)-1H-1,2,3-triazol-1-yl)phenyl)pyridine* (**26e**)

^1^H NMR (400 MHz, Chloroform-*d*) δ 8.68 (d, *J* = 2.2 Hz, 1H), 8.57 (d, *J* = 4.9 Hz, 1H), 7.94 (s, 1H), 7.72 (dt, *J* = 8.0, 2.0 Hz, 1H), 7.65 (d, *J* = 8.1 Hz, 2H), 7.42 (d, *J* = 8.1 Hz, 2H), 7.36 – 7.28 (m, 3H), 7.05 (d, *J* = 8.6 Hz, 1H), 3.89 (s, 3H). ^13^C NMR (101 MHz, CDCl_3_) δ 157.37, 150.15, 148.79, 136.81, 136.49, 133.86, 130.54, 129.52, 129.02, 128.40, 127.54, 126.31, 126.14, 126.11, 126.07, 126.03, 123.21, 111.91, 56.09. HRMS-ESI: calcd. for C_21_H_16_F_3_N_4_O, 397.1271 [M+1]+; found 397.1271. Purity 90.2%

*4-(2-methoxy-5-(5-(4-(trifluoromethyl)phenyl)-1H-1,2,3-triazol-1-yl)phenyl)pyridine* (**26f**)

^1^H NMR (400 MHz, Chloroform-*d*) δ 8.61 (s, 2H), 7.94 (s, 1H), 7.66 (d, *J* = 8.0 Hz, 2H), 7.41 (d, *J* = 8.1 Hz, 2H), 7.37 – 7.28 (m, 4H), 7.11 – 6.96 (m, 1H), 3.90 (s, 3H). ^13^C NMR (101 MHz, CDCl_3_) δ 157.28, 149.80, 144.51, 136.46, 133.87, 130.57, 130.56, 129.50, 129.08, 128.90, 127.35, 126.74, 126.16, 126.12, 126.08, 126.05, 112.09, 56.12. HRMS-ESI: calcd. for C_21_H_16_F_3_N_4_O, 397.1271 [M+1]+; found 397.1277. Purity 93.8%

*4-(2-methoxy-5-(5-(4-(trifluoromethyl)phenyl)-1H-1,2,3-triazol-1-yl)phenyl)pyridazine* (**26g**)

^1^H NMR (400 MHz, Chloroform-*d*) δ 9.34 (dd, *J* = 2.4, 1.2 Hz, 1H), 9.21 (dd, *J* = 5.4, 1.2 Hz, 1H), 7.95 (s, 1H), 7.72 – 7.61 (m, 2H), 7.52 (dd, *J* = 5.4, 2.4 Hz, 1H), 7.45 (d, *J* = 2.6 Hz, 1H), 7.43 – 7.39 (m, 2H), 7.37 (dd, *J* = 8.8, 2.7 Hz, 1H), 7.09 (d, *J* = 8.9 Hz, 1H), 3.93 (s, 3H). ^13^C NMR (101 MHz, CDCl_3_) δ 157.57, 151.67, 151.33, 136.52, 135.31, 134.03, 130.39, 129.89, 129.07, 127.91, 127.24, 126.26, 126.23, 126.19, 126.15, 125.95, 125.23, 112.35, 56.29. HRMS-ESI: calcd. for C_20_H_16_F_3_N_5_O, 398.1223 [M+1]+; found 398.1218. Purity 91.3%

*1-(3-(3,6-dihydro-2H-pyran-4-yl)-4-methoxyphenyl)-5-(4-(trifluoromethyl)phenyl)-1H-1,2,3-triazole* (**26h**)

^1^H NMR (400 MHz, Chloroform-*d*) δ 7.92 (s, 1H), 7.63 (d, *J* = 8.1 Hz, 2H), 7.45 – 7.34 (m, 2H), 7.20 (dd, *J* = 8.7, 2.7 Hz, 1H), 7.14 (d, *J* = 2.7 Hz, 1H), 6.91 (d, *J* = 8.7 Hz, 1H), 5.76 (tt, *J* = 2.9, 1.6 Hz, 1H), 4.25 (q, *J* = 2.7 Hz, 2H), 3.87 (s, 3H), 3.85 (d, *J* = 5.4 Hz, 1H), 2.43 (ttd, *J* = 5.5, 2.7, 1.6 Hz, 2H). ^13^C NMR (101 MHz, CDCl_3_) δ 157.66, 136.42, 133.73, 133.46, 132.40, 129.08, 129.02, 126.39, 126.15, 126.03, 126.00, 125.96, 125.92, 125.08, 111.37, 65.71, 64.45, 55.88, 28.58. HRMS-ESI: calcd. for C_21_H_19_F_3_N_3_O_2_, 402.1424 [M+1]+; found 402.1423. Purity 94.8%

*1-(3-(3,4-dihydro-2H-pyran-5-yl)-4-methoxyphenyl)-5-(4-(trifluoromethyl)phenyl)-1H-1,2,3-triazole* (**26i**)

^1^H NMR (400 MHz, Chloroform-*d*) δ 7.92 (s, 1H), 7.62 (d, *J* = 8.2 Hz, 2H), 7.40 – 7.33 (m, 2H), 7.12 (d, *J* = 2.1 Hz, 1H), 7.10 (d, *J* = 2.7 Hz, 1H), 6.88 (d, *J* = 8.3 Hz, 1H), 6.77 (t, *J* = 1.6 Hz, 1H), 4.04 – 3.99 (m, 2H), 3.88 (s, 3H), 2.29 (td, *J* = 6.3, 1.5 Hz, 2H), 2.02 – 1.89 (m, 2H). ^13^C NMR (101 MHz, CDCl_3_) δ 157.83, 144.71, 136.28, 133.57, 130.58, 128.98, 128.84, 126.67, 125.88, 125.84, 125.80, 125.77, 125.48, 123.64, 114.74, 111.10, 110.28, 65.55, 55.72, 23.41, 22.30. HRMS-ESI: calcd. for C_21_H_19_F_3_N_3_O_2_, 402.1424 [M+1]+; found 402.1414. Purity 90.7%

*tert-butyl 4-(2-methoxy-5-(5-(4-(trifluoromethyl)phenyl)-1H-1,2,3-triazol-1-yl)phenyl)-3,6-dihydropyridine-1(2H)-carboxylate* (**26j**)

^1^H NMR (400 MHz, Chloroform-*d*) δ 7.90 (s, 1H), 7.61 (d, *J* = 8.1 Hz, 2H), 7.36 (d, *J* = 8.1 Hz, 2H), 7.23 – 7.07 (m, 2H), 6.89 (d, *J* = 8.7 Hz, 1H), 5.63 (s, 1H), 3.97 (q, *J* = 2.8 Hz, 2H), 3.85 (s, 3H), 3.54 (t, *J* = 5.5 Hz, 2H), 2.42 (s, 2H), 1.48 (s, 9H).

*1-(4-methoxy-3-(2,2,6,6-tetramethyl-3,6-dihydro-2H-pyran-4-yl)phenyl)-5-(4-(trifluoromethyl)phenyl)-1H-1,2,3-triazole* (**26k**)

^1^H NMR (400 MHz, Chloroform-*d*) δ 7.93 (d, *J* = 2.3 Hz, 1H), 7.64 (d, *J* = 8.0 Hz, 2H), 7.39 (d, *J* = 8.0 Hz, 1H), 7.22 (s, 1H), 7.09 (d, *J* = 2.7 Hz, 1H), 6.90 (d, *J* = 8.7 Hz, 1H), 5.56 (s, 1H), 3.86 (s, 3H), 3.65 (s, 3H), 2.25 (s, 1H), 1.25 (s, 12H). ^13^C NMR (101 MHz, CDCl_3_) δ 133.64, 129.38, 129.07, 128.95, 128.78, 128.18, 127.72, 127.66, 126.67, 126.00, 125.91, 125.86, 125.83, 125.26, 124.68, 114.74, 55.68, 55.62, 30.16, 29.72, 29.00. HRMS-ESI: calcd. for C_25_H_27_F_3_N_3_O_2_, 458.2050 [M+1]+; found 458.2043. Purity 92.0%

*1-(4',6-dimethoxy-2',3',4',5'-tetrahydro-[1,1'-biphenyl]-3-yl)-5-(4-(trifluoromethyl)phenyl)-1H-1,2,3-triazole* (**26l**)

^1^H NMR (400 MHz, Chloroform-*d*) δ 7.92 (s, 1H), 7.62 (d, *J* = 8.2 Hz, 2H), 7.37 (d, *J* = 8.1 Hz, 2H), 7.17 (dd, *J* = 8.7, 2.7 Hz, 1H), 7.11 (d, *J* = 2.7 Hz, 1H), 6.88 (d, *J* = 8.7 Hz, 1H), 5.55 (ddt, *J* = 4.8, 3.3, 1.6 Hz, 1H), 3.86 (s, 3H), 3.52 (dddd, *J* = 10.1, 8.1, 5.3, 3.0 Hz, 1H), 3.39 (s, 3H), 2.55 – 2.33 (m, 3H), 2.20 – 2.06 (m, 1H), 1.99 (ddt, *J* = 10.8, 6.8, 2.6 Hz, 1H), 1.70 (dtd, *J* = 12.6, 9.0, 5.8 Hz, 1H). ^13^C NMR (101 MHz, CDCl_3_) δ 157.57, 136.39, 136.02, 133.93, 133.63, 130.73, 130.72, 128.98, 128.93, 126.57, 125.98, 125.94, 125.90, 125.86, 125.21, 124.74, 111.17, 75.26, 55.86, 31.81, 29.83, 27.63, 26.81. HRMS-ESI: calcd. for C_23_H_22_F_3_N_3_O_2_, 452.1557 [M+Na]+; found 452.1565. Purity 93.0%

*1-(3-(4,5-dihydrofuran-3-yl)-4-methoxyphenyl)-5-(4-(trifluoromethyl)phenyl)-1H-1,2,3-triazole* (**26m**)

^1^H NMR (400 MHz, Chloroform-*d*) δ 7.92 (s, 1H), 7.61 (d, *J* = 8.2 Hz, 2H), 7.44 – 7.33 (m, 3H), 7.07 – 6.95 (m, 2H), 6.89 (d, *J* = 8.6 Hz, 1H), 4.41 (t, *J* = 9.5 Hz, 2H), 3.96 (s, 3H), 2.82 (td, *J* = 9.6, 1.9 Hz, 2H). ^13^C NMR (101 MHz, CDCl_3_) δ 157.01, 148.04, 136.45, 133.73, 129.32, 128.94, 126.02, 125.98, 125.94, 125.90, 125.11, 123.09, 122.19, 110.44, 110.27, 69.35, 55.77, 31.29. HRMS-ESI: calcd. for C_20_H_17_F_3_N_3_O_2_, 388.1267 [M+1]+; found 388.1265. Purity 91.0%

*1-(3-(2,5-dihydrofuran-3-yl)-4-methoxyphenyl)-5-(4-(trifluoromethyl)phenyl)-1H-1,2,3-triazole* (**26n**)

^1^H NMR (400 MHz, Chloroform-*d*) δ 7.93 (s, 1H), 7.63 (d, *J* = 8.2 Hz, 2H), 7.40 – 7.30 (m, 2H), 7.20 – 7.10 (m, 2H), 6.96 (d, *J* = 8.6 Hz, 1H), 6.54 (p, *J* = 1.9 Hz, 1H), 4.96 – 4.78 (m, 3H), 3.94 (s, 3H). ^13^C NMR (101 MHz, CDCl_3_) δ 158.12, 136.48, 133.83, 133.69, 129.13, 128.96, 126.76, 126.09, 126.05, 126.02, 125.98, 125.35, 125.15, 123.15, 111.52, 77.34, 76.96, 76.22, 29.83. HRMS-ESI: calcd. for C_20_H_17_F_3_N_3_O_2_, 388.1267 [M+1]+; found 388.1259. Purity 91.1%

*1-(4-methoxy-3-(tetrahydro-2H-pyran-4-yl)phenyl)-5-(4-(trifluoromethyl)phenyl)-1H-1,2,3-triazole* (**27**)

^1^H NMR (400 MHz, Chloroform-*d*) δ 7.93 (s, 1H), 7.61 (d, *J* = 8.2 Hz, 2H), 7.42 – 7.31 (m, 2H), 7.20 (dd, *J* = 8.7, 2.6 Hz, 1H), 7.06 (d, *J* = 2.6 Hz, 1H), 6.90 (d, *J* = 8.7 Hz, 1H), 4.06 – 3.91 (m, 2H), 3.88 (s, 2H), 3.51 (td, *J* = 11.7, 2.3 Hz, 2H), 3.18 (tt, *J* = 11.8, 3.8 Hz, 1H), 1.63 (ddt, *J* = 11.1, 4.4, 2.1 Hz, 2H), 1.59 – 1.46 (m, 2H). ^13^C NMR (101 MHz, CDCl_3_) δ 157.57, 136.42, 135.52, 133.71, 129.21, 129.02, 126.00, 125.96, 125.93, 125.89, 124.07, 124.05, 110.89, 68.46, 55.81, 34.32, 32.42. HRMS-ESI: calcd. for C_21_H_21_F_3_N_3_O_2_, 404.1580 [M+1]+; found 404.1591. Purity 92.1%

*1-(4-methoxy-3-(tetrahydrofuran-3-yl)phenyl)-5-(4-(trifluoromethyl)phenyl)-1H-1,2,3-triazole* (**28**)

^1^H NMR (400 MHz, Chloroform-*d*) δ 7.85 (s, 1H), 7.55 (d, *J* = 8.0 Hz, 2H), 7.31 – 7.26 (m, 2H), 7.17 – 7.04 (m, 3H), 6.83 (d, *J* = 8.7 Hz, 2H), 3.97 (dd, *J* = 8.4, 7.1 Hz, 2H), 3.82 (s, 3H), 3.61 – 3.54 (m, 3H), 2.29 (t, *J* = 7.5 Hz, 1H), 2.24 – 2.09 (m, 2H), 1.73 (dq, *J* = 12.5, 7.1 Hz, 2H). ^13^C NMR (101 MHz, CDCl_3_) δ 164.53, 133.61, 129.05, 128.93, 125.87, 125.84, 125.80, 125.77, 124.28, 124.26, 124.25, 124.24, 117.95, 110.70, 106.54, 72.99, 67.81, 55.74, 37.90, 29.72. HRMS-ESI: calcd. for C_20_H_18_F_3_N_3_O_2_, 412.1243 [M+Na]+; found 412.1234. Purity 90.8%

*4-(2-methoxy-5-(5-(4-(trifluoromethyl)phenyl)-1H-1,2,3-triazol-1-yl)phenyl)-1,2,3,6-tetrahydropyridine* (**29**)

^1^H NMR (400 MHz, Chloroform-*d*) δ 7.91 (s, 1H), 7.62 (d, *J* = 7.9 Hz, 2H), 7.37 (d, *J* = 8.1 Hz, 2H), 7.24 – 7.04 (m, 2H), 6.89 (d, *J* = 8.7 Hz, 1H), 5.68 (s, 1H), 3.86 (s, 3H), 3.59 – 3.45 (m, 2H), 3.12 (q, *J* = 7.4, 5.7 Hz, 2H), 2.47 (s, 2H). ^13^C NMR (101 MHz, CDCl_3_) δ 157.57, 136.41, 134.96, 133.70, 132.75, 131.06, 130.68, 129.04, 129.01, 126.35, 125.98, 125.95, 125.20, 124.84, 111.32, 55.87, 44.15, 42.37, 27.84. HRMS-ESI: calcd. for C_21_H_20_F_3_N_4_O, 401.1584 [M+1]+; found 401.1586. Purity 90.8%

*4-(2-methoxy-5-(5-(4-(trifluoromethyl)phenyl)-1H-1,2,3-triazol-1-yl)phenyl)-3,6-dihydropyridine-1(2H)-carbaldehyde* (**30**)

^1^H NMR (400 MHz, Chloroform-*d*) δ 8.12 (s, 1H), 7.93 (s, 1H), 7.63 (d, *J* = 8.2 Hz, 2H), 7.38 (d, *J* = 8.1 Hz, 2H), 7.21 (ddd, *J* = 7.9, 5.1, 2.7 Hz, 1H), 7.12 (dd, *J* = 6.4, 2.7 Hz, 1H), 6.91 (dd, *J* = 8.8, 1.8 Hz, 1H), 5.65 (s, 1H), 4.11 (q, *J* = 2.8 Hz, 1H), 4.03 – 3.91 (m, 1H), 3.87 (d, *J* = 2.0 Hz, 3H), 3.71 (t, *J* = 5.8 Hz, 1H), 3.68 – 3.60 (m, 1H), 3.55 (t, *J* = 5.7 Hz, 1H), 2.52 (d, *J* = 7.6 Hz, 2H), 2.35 (t, *J* = 7.5 Hz, 1H), 1.62 (q, *J* = 7.3 Hz, 2H). ^13^C NMR (101 MHz, CDCl_3_) δ 162.10, 161.65, 157.79, 134.88, 134.09, 132.58, 129.37, 126.59, 126.39, 126.36, 126.32, 126.27, 125.80, 125.75, 125.72, 123.55, 123.18, 111.69, 56.22, 43.20, 40.66, 30.15. HRMS-ESI: calcd. for C_22_H_20_F_3_N_4_O_2_, 429.1533 [M+1]+; found 429.1540. Purity 90.5%

*1-(4-(2-methoxy-5-(5-(4-(trifluoromethyl)phenyl)-1H-1,2,3-triazol-1-yl)phenyl)-3,6-dihydropyridin-1(2H)-yl)ethan-1-one* (**31**)

^1^H NMR (400 MHz, Chloroform-*d*) δ 7.92 (s, 1H), 7.63 (d, *J* = 8.2 Hz, 2H), 7.38 (d, *J* = 8.1 Hz, 2H), 7.18 (ddd, *J* = 14.7, 8.5, 2.7 Hz, 1H), 7.12 (d, *J* = 2.7 Hz, 1H), 6.90 (dd, *J* = 8.7, 1.3 Hz, 1H), 5.67 (dtt, *J* = 20.5, 3.3, 1.5 Hz, 1H), 4.15 (q, *J* = 2.8 Hz, 1H), 4.05 (q, *J* = 2.8 Hz, 1H), 3.87 (d, *J* = 1.4 Hz, 3H), 3.73 (t, *J* = 5.7 Hz, 1H), 3.58 (t, *J* = 5.6 Hz, 1H), 2.54 – 2.38 (m, 2H), 2.13 (d, *J* = 8.5 Hz, 3H). ^13^C NMR (101 MHz, CDCl_3_) δ 169.53, 169.41, 157.49, 136.40, 136.27, 134.13, 133.74, 129.02, 126.22, 126.15, 126.04, 126.00, 125.93, 125.33, 125.27, 124.71, 122.97, 111.33, 55.88, 43.43, 42.13, 29.21, 21.69. HRMS-ESI: calcd. for C_23_H_22_F_3_N_4_O_2_, 443.1689 [M+1]+; found 443.1682. Purity 95.6%

*4-(2-methoxy-5-(5-(4-(trifluoromethyl)phenyl)-1H-1,2,3-triazol-1-yl)phenyl)morpholine* (**32**)

^1^H NMR (400 MHz, Chloroform-*d*) δ 12.11 (s, 1H), 7.92 (d, *J* = 2.7 Hz, 1H), 7.61 (d, *J* = 8.1 Hz, 2H), 7.36 (d, *J* = 8.0 Hz, 2H), 7.27 (d, *J* = 4.0 Hz, 1H), 7.26 (d, *J* = 1.9 Hz, 2H), 7.02 – 6.86 (m, 2H), 3.86 (d, *J* = 2.6 Hz, 3H), 3.10 (q, *J* = 7.3 Hz, 3H), 1.57 (s, 1H), 1.41 (t, *J* = 7.2 Hz, 4H). ^13^C NMR (101 MHz, CDCl_3_) δ 160.59, 136.60, 133.87, 131.49, 130.70, 129.34, 129.00, 126.89, 126.16, 126.12, 126.08, 126.05, 114.96, 55.84, 46.00, 8.86. HRMS-ESI: calcd. for C_19_H_20_F_5_N_3_O, 401.1521 [M+1]+; found 401.1541. Purity 96.8%

*2-oxaspiro[3.5]non-6-en-7-yl trifluoromethanesulfonate* (**34**)

^1^H NMR (400 MHz, Chloroform-*d*) δ 5.70 (tt, *J* = 3.9, 1.6 Hz, 1H), 4.47 (d, *J* = 6.0 Hz, 2H), 4.43 (d, *J* = 6.0 Hz, 2H), 2.53 (dt, *J* = 4.4, 2.6 Hz, 2H), 2.40 (tq, *J* = 6.5, 2.4 Hz, 2H), 2.08 (t, *J* = 6.5 Hz, 2H).

*1-(4-methoxy-3-(4,4,5,5-tetramethyl-1,3,2-dioxaborolan-2-yl)phenyl)-5-(4-(trifluoromethyl)phenyl)-1H-1,2,3-triazole* (**35**)

^1^H NMR (400 MHz, Chloroform-*d*) δ 7.84 (s, 1H), 7.63 (d, *J* = 2.8 Hz, 1H), 7.52 (d, *J* = 8.2 Hz, 2H), 7.29 (d, *J* = 8.1 Hz, 2H), 7.24 – 7.16 (m, 1H), 6.91 – 6.72 (m, 1H), 3.81 (s, 2H), 2.10 (s, 2H), 1.17 (s, 15H).

*1-(4-methoxy-3-(2-oxaspiro[3.5]non-6-en-7-yl)phenyl)-5-(4-(trifluoromethyl)phenyl)-1H-1,2,3-triazole* (**37a**)

^1^H NMR (400 MHz, Chloroform-*d*) δ 7.86 (d, *J* = 7.0 Hz, 1H), 7.55 (d, *J* = 8.2 Hz, 2H), 7.30 (d, *J* = 8.1 Hz, 2H), 7.08 (dd, *J* = 8.6, 2.7 Hz, 1H), 7.02 (d, *J* = 2.7 Hz, 1H), 6.80 (d, *J* = 8.7 Hz, 1H), 5.51 (s, 1H), 4.48 – 4.33 (m, 3H), 3.77 (d, *J* = 8.9 Hz, 2H), 3.58 (q, *J* = 8.3, 5.9 Hz, 2H), 2.44 – 2.35 (m, 2H), 2.31 (s, 2H), 1.91 (t, *J* = 6.3 Hz, 2H). ^13^C NMR (101 MHz, CDCl_3_) δ 157.53, 136.42, 136.40, 133.80, 133.70, 133.69, 129.03, 126.56, 126.01, 125.97, 125.93, 125.89, 125.43, 124.83, 112.70, 111.21, 82.81, 55.90, 37.97, 35.99, 31.13, 25.48. HRMS-ESI: calcd. for C_24_H_23_F_3_N_3_O_2_, 442.1737 [M+1]+; found 442.1745. Purity 91.1%

*5-(2-methoxy-5-(5-(4-(trifluoromethyl)phenyl)-1H-1,2,3-triazol-1-yl)phenyl)pyrimidine* (**37b**)

^1^H NMR (400 MHz, Chloroform-*d*) δ 9.18 (s, 1H), 8.83 (s, 2H), 7.95 (s, 1H), 7.66 (d, *J* = 8.1 Hz, 2H), 7.48 – 7.38 (m, 3H), 7.35 (dd, *J* = 8.8, 2.7 Hz, 1H), 7.07 (d, *J* = 8.8 Hz, 1H), 3.91 (s, 3H). ^13^C NMR (101 MHz, CDCl_3_) δ 157.79, 157.72, 157.39, 156.91, 156.81, 134.00, 130.75, 130.72, 130.44, 130.41, 129.81, 129.03, 127.26, 127.15, 126.24, 126.20, 126.16, 126.13, 112.10, 56.23. HRMS-ESI: calcd. for C_20_H_15_F_3_N_5_O, 398.1223 [M+1]+; found 398.1211. Purity 93.2%

*triisopropyl((4-(4,4,5,5-tetramethyl-1,3,2-dioxaborolan-2-yl)cyclohex-3-en-1-yl)oxy)silane* (**39**)

^1^H NMR (400 MHz, Chloroform-*d*) δ 6.44 (dt, *J* = 4.7, 2.1 Hz, 1H), 3.94 (dddd, *J* = 10.7, 8.3, 5.3, 3.3 Hz, 1H), 2.47 – 2.26 (m, 2H), 2.23 – 2.00 (m, 2H), 1.86 (dddt, *J* = 12.6, 5.5, 3.7, 1.8 Hz, 1H), 1.54 (ddt, *J* = 12.5, 10.2, 5.0 Hz, 1H), 1.25 (s, 3H), 1.05 (q, *J* = 2.7, 2.2 Hz, 18H).

*1-(6-methoxy-4'-((triisopropylsilyl)oxy)-2',3',4',5'-tetrahydro-[1,1'-biphenyl]-3-yl)-5-(4-(trifluoromethyl)phenyl)-1H-1,2,3-triazole* (**40**)

^1^H NMR (400 MHz, Chloroform-*d*) δ 7.92 (s, 1H), 7.62 (d, *J* = 8.1 Hz, 2H), 7.37 (d, *J* = 8.1 Hz, 2H), 7.16 (dd, *J* = 8.7, 2.7 Hz, 1H), 7.10 (d, *J* = 2.7 Hz, 1H), 6.87 (d, *J* = 8.7 Hz, 1H), 5.53 (dq, *J* = 3.7, 1.7 Hz, 1H), 4.06 (dddd, *J* = 10.0, 8.1, 5.1, 3.1 Hz, 1H), 3.85 (s, 3H), 2.50 – 2.32 (m, 3H), 2.19 – 2.08 (m, 1H), 1.95 – 1.83 (m, 1H), 1.73 (dtd, *J* = 12.5, 9.1, 5.5 Hz, 1H), 1.06 (d, *J* = 3.4 Hz, 15H), 1.04 (s, 4H).

*2'-methoxy-5'-(5-(4-(trifluoromethyl)phenyl)-1H-1,2,3-triazol-1-yl)-2,3,4,5-tetrahydro-[1,1'-biphenyl]-4-ol* (**41**)

^1^H NMR (400 MHz, Chloroform-*d*) δ 7.92 (s, 1H), 7.67 – 7.58 (m, 2H), 7.42 – 7.33 (m, 2H), 7.17 (dd, *J* = 8.7, 2.7 Hz, 1H), 7.11 (d, *J* = 2.7 Hz, 1H), 6.88 (d, *J* = 8.7 Hz, 1H), 5.54 (ddt, *J* = 4.9, 3.3, 1.4 Hz, 1H), 4.03 (s, 1H), 3.86 (s, 3H), 2.55 – 2.35 (m, 3H), 2.14 (ddt, *J* = 15.3, 6.5, 2.6 Hz, 1H), 1.93 (dddd, *J* = 11.4, 9.8, 5.7, 2.9 Hz, 1H), 1.80 – 1.67 (m, 1H). ^13^C NMR (101 MHz, CDCl_3_) δ 157.57, 136.41, 135.97, 133.92, 133.66, 131.38, 131.06, 130.73, 129.01, 128.97, 126.58, 126.00, 125.97, 125.93, 125.89, 124.94, 124.83, 111.19, 66.50, 55.89, 34.85, 31.16. HRMS-ESI: calcd. for C_22_H_21_F_3_N_3_O_2_, 416.1580 [M+1]+; found 416.1578. Purity 92.0%

*1-(6-methoxy-4'-(trifluoromethoxy)-2',3',4',5'-tetrahydro-[1,1'-biphenyl]-3-yl)-5-(4-(trifluoromethyl)phenyl)-1H-1,2,3-triazole* (**42**)

^1^H NMR (400 MHz, Chloroform-*d*) δ 7.88 (s, 1H), 7.58 (d, *J* = 8.1 Hz, 2H), 7.33 (d, *J* = 8.1 Hz, 2H), 7.13 (dd, *J* = 8.8, 2.7 Hz, 1H), 7.05 (d, *J* = 2.7 Hz, 1H), 6.86 (d, *J* = 8.7 Hz, 1H), 5.49 (dq, *J* = 4.6, 2.1 Hz, 1H), 3.95 (qd, *J* = 8.9, 8.1, 4.0 Hz, 1H), 3.82 (s, 3H), 2.50 – 2.32 (m, 3H), 2.07 (ddt, *J* = 14.7, 6.9, 2.7 Hz, 1H), 1.94 – 1.83 (m, 1H), 1.67 (dtd, *J* = 12.5, 8.8, 6.4 Hz, 1H). ^13^C NMR (101 MHz, CDCl_3_) δ 157.60, 135.75, 133.91, 131.39, 131.07, 130.38, 128.69, 126.46, 125.94, 125.90, 125.87, 125.83, 125.05, 124.77, 122.36, 121.15, 117.97, 111.20, 66.24, 55.79, 50.22, 34.51, 30.97, 26.69. HRMS-ESI: calcd. for C_23_H_20_F_6_N_3_O_2_, 484.1454 [M+1]+; found 484.1449. Purity 96.1%

*3-(1-(3-iodo-4-methoxyphenyl)-1H-1,2,3-triazol-5-yl)pyridine* (**43**)

^1^H NMR (400 MHz, Chloroform-*d*) δ 8.57 (dd, *J* = 4.8, 1.6 Hz, 1H), 8.49 (d, *J* = 2.3 Hz, 1H), 7.87 (s, 1H), 7.57 (d, *J* = 2.5 Hz, 1H), 7.46 (dt, *J* = 8.0, 2.0 Hz, 1H), 7.25 (ddd, *J* = 8.1, 4.9, 0.9 Hz, 1H), 7.16 (dd, *J* = 8.8, 2.6 Hz, 1H), 6.89 – 6.82 (m, 1H), 3.88 (s, 3H).

*3-(1-(3-(3,6-dihydro-2H-pyran-4-yl)-4-methoxyphenyl)-1H-1,2,3-triazol-5-yl)pyridine* (**44**)

^1^H NMR (400 MHz, Chloroform-*d*) δ 8.62 (dd, *J* = 4.9, 1.7 Hz, 1H), 8.55 (d, *J* = 2.2 Hz, 1H), 7.94 (s, 1H), 7.55 (dt, *J* = 7.9, 2.0 Hz, 1H), 7.31 (ddd, *J* = 8.0, 4.9, 0.9 Hz, 1H), 7.21 (dd, *J* = 8.6, 2.7 Hz, 1H), 7.16 (d, *J* = 2.7 Hz, 1H), 6.91 (d, *J* = 8.7 Hz, 1H), 5.79 (tt, *J* = 2.9, 1.6 Hz, 1H), 4.26 (q, *J* = 2.7 Hz, 2H), 3.87 (s, 3H), 3.85 (d, *J* = 5.4 Hz, 1H), 2.44 (tdd, *J* = 5.4, 4.4, 2.5 Hz, 2H). ^13^C NMR (101 MHz, CDCl_3_) δ 157.76, 150.31, 149.22, 135.80, 134.77, 133.62, 133.46, 132.58, 128.99, 126.36, 126.24, 125.21, 123.62, 123.48, 111.45, 65.74, 64.46, 55.90, 28.59. HRMS-ESI: calcd. for C_19_H_19_N_4_O_2_, 335.1503 [M+1]+; found 335.1504. Purity 94.2%

**NMR data**

**26f**

**26h**

**HPLC trace purity data**

**26f** purity= 97.5%

**26h** purity= 93.3%

Supplementary Figure 1. SK-Br-3 cells were treated with compound for 24 hours, then subject to Aha1 immunoprecipitation (IP), where Hsp90$\alpha$ co-IP was evaluated by western blot. Inputs were loaded on a separate gel for Western blot.

Supplementary Figure 2. Monolayer integrity was evaluated by Lucifer Yellow (LY) measurement. MDCK-MFR1 cells were grown into confluent monolayers on 24 well plate cell culture inserts (Thermo Scientific), where 60 µM LY was added to the apical chamber of wells containing monolayers intensity (monolayer, n=12) and compared to the 60 µM LY solution (empty well, n=6). The monolayer allowed <5% of the LY to permeate indicating monolayer integrity. Average values and standard deviations are shown in the above figure.
